# Resource management as a conservation tool to impact genetic diversity through mating patterns in wild populations

**DOI:** 10.1002/eap.70226

**Published:** 2026-04-02

**Authors:** Noa Yaffa Kan‐Lingwood, Liran Sagi, Alan R. Templeton, Naama Shahar, Ariel Altman, Nurit Gordon, Daniel I. Rubenstein, Amos Bouskila, Shirli Bar‐David

**Affiliations:** ^1^ Mitrani Department of Desert Ecology Ben‐Gurion University of the Negev, The Swiss Institute for Dryland Environmental & Energy Research, Blaustein Institutes for Desert Research, Sde Boker Campus Midreshet Ben‐Gurion Israel; ^2^ Life Science Department Ben‐Gurion University of the Negev Beer‐Sheva Israel; ^3^ Department of Biology Washington University St. Louis Missouri USA; ^4^ Department of Ecology and Evolutionary Biology Princeton University Princeton New Jersey USA

**Keywords:** adaptive management, arid ecosystems, *Equus hemionus*, genetic diversity, parentage analysis, parentage simulations, population genetics, resource defense polygyny, resource management, water sources

## Abstract

The distribution of resources influences interactions in wild populations by affecting movement, space‐use patterns, and, as a result, mating systems. Limited resources may reduce encounters between potential breeders, reducing the number and variety of individuals contributing to the population's gene pool. This can impact the variance effective population size (*N*_ev_), an important indicator of genetic drift and genetic diversity in populations. Despite its importance, the relationship between resource distribution and genetic diversity has received limited attention in the practical management of genetically vulnerable populations. Here, we provide empirical evidence that the number of reproducing males (adult males identified as sires by parentage analysis from foal genotypes) and *N*_ev_ can be affected by resource management in the population of the Asiatic wild ass (*Equus hemionus*) in the Negev Desert, Israel, using water sources. This population, characterized by strong polygyny, has experienced declining genetic diversity. Following an intervention to increase the number of water sources from one to three during May 2020, we monitored the population using noninvasive genetic methods and direct observations. We collected 864 fecal samples from adult males, females, and foals, genotyped the DNA across 535 single nucleotide polymorphisms (SNPs), and conducted a parentage analysis. The results showed an increase in the proportion of total reproducing males out of all adult males in the population from 16%–18% to 42%–48%, with a significant rise in reproducing males in 2020 that had not been sires in 2019, from 31.2% to 73.8% (*Z* = −2.877, *p* = 0.002) before and after the management, respectively. Spatial analyses indicated a higher presence of reproducing males near the new water sources. These findings demonstrate how resource management can impact *N*_ev_ in the short term and, therefore, potentially influence genetic diversity in the long term. We suggest a management framework targeting genetic diversity within an adaptive management approach and discuss its relevance and applicability for other systems and types of resources.

## INTRODUCTION

Resource distribution plays a central role in shaping wildlife behavior, influencing movement patterns (Bakner et al., 2023; Tanner et al., 2015), social and mating structure (Halliwell et al., 2016), and reproductive opportunities (Bowyer et al., 2020; Cutting et al., 2021; Lindström, 2001). The availability of essential resources, such as food, water, and shelter, shapes individuals' space‐use patterns and, as a result, how they interact, ultimately shaping their social strategies and reproductive success (Carvalho et al., 2017). Therefore, due to the influence of resources on mating opportunities, their distribution may influence genetic diversity and population structure by impacting which and how many individuals out of all individuals in the population contribute their alleles to the population's gene pool.

For example, when resource patches (such as water or food) are geographically distant from one another due to physical barriers, it may cause individuals of a specific population to concentrate around the resources, thereby restricting subpopulation connectivity and encounters between potential breeders (Albert & García‐Navas, 2022; Burkhart et al., 2017; Carvalho et al., 2017). This process can reduce gene flow between subpopulations, increase inbreeding events, accelerate genetic drift, and impact genetic structure (Templeton, 2021). Additionally, in systems where competition for vital resources drives social and mating interactions (Albert & García‐Navas, 2022; Bowyer et al., 2020; Wilson et al., 2011), intense competition over or in proximity to limited resources may restrict reproductive opportunities to a subset of potential breeders and, as a result, reduce the variance effective population size (*N*_ev_) by skewing the proportional representation of ancestors in future generations (Ficetola et al., 2010; Parreira & Chikhi, 2015; Templeton, 2021). *N*_ev_ reflects the size that measures the impact of genetic drift on allele frequencies, including loss and fixation, therefore reflecting genetic diversity loss (Waples, 2025). In contrast to the inbreeding effective size that looks backwards (i.e., inbreeding effective size is strongly influenced by the number of ancestors of the current population), *N*_ev_ is forward‐looking and is primarily influenced by the number of successful parents who are passing on genes to the next generation. Hence, the forward‐looking *N*_ev_ is an indicator of the long‐term genetic resilience of the population. This forward‐looking property is of fundamental importance in conservation biology, so *N*_ev_ is the most commonly used effective population size indicator to assess genetic diversity in wild populations (Allendorf et al., 2024; Fedorca et al., 2024; Ryman et al., 2019; Templeton, 2021). Despite this connection, limited research has focused on the relationship between resource distribution and genetic diversity and how resources can be practically managed to support genetically vulnerable populations.

Water is a critical resource for many wildlife species, especially in arid environments. Water availability and distribution may impact a variety of aspects related to species' needs and populations' processes (e.g., drinking [Sutherland et al., 2018], thermal regulation [Sawaya et al., 2016], space‐use patterns [Amoroso et al., 2020; Smit et al., 2007; Tanner et al., 2015], demography [Ovalle‐Rivera et al., 2020; Thor et al., 2023], and geographical distribution [Redfern et al., 2005]). Moreover, considerable temporal and spatial water fluctuations are expected under climate change (Seddon et al., 2020). Recent studies have increasingly reported alterations in water availability, flow, and evaporation rates worldwide (Cantonati et al., 2020; Lawler, 2009), making freshwater preservation relevant not only in arid and semiarid environments but also in various types of habitats on a global scale (Seddon et al., 2020). The management of water sources was shown to affect species diversity (Ovalle‐Rivera et al., 2020; Samways et al., 2020), distribution and foraging (Rich et al., 2019), behavior (Mohr et al., 2022; Tanner et al., 2015), habitat use (Amoroso et al., 2020), demography (Thor et al., 2023), and breeding (Elhassan et al., 2021). However, less attention has been given to the potential genetic impacts of water source management.

Here, we examined how changing the locations and increasing the number of water sources impact the number of reproducing males (i.e., adult males that produce foals—individuals that were born during the particular sampling year—as detected by parentage analysis based on foals' genotypes) out of all adult males in the population (i.e., the proportion of males that reproduce) and *N*_ev_ in the Asiatic wild ass (*Equus hemionus*) population in the Negev Desert, Israel.

The population possesses a well‐documented resource defense polygyny mating system (Emlen & Oring, 1977), also called territorial polygyny. In this system, a few solitary males gain a reproductive advantage by defending areas near water sources and vegetation, which attract females (Renan et al., 2018; Saltz et al., 2000). These territorial males represent the breeding portion out of all sexually mature adult males of the population. In contrast, most adult males remain non‐territorial “bachelors” that do not get the opportunity to reproduce. Territorial behavior and space use are most evident during the breeding season (May–September), when females are in estrus (Giotto et al., 2015; Saltz et al., 2000). During this time, lactating females need daily access to water (Nowzari et al., 2013; Rubenstein et al., 2007). Consequently, females spend extended periods within territories near water, where mating also takes place (Saltz et al., 2000).

Thus, changing the availability of water sources was expected to allow more bachelor adult males to establish territories near the new water sources and increase their chances of becoming territorial, reproducing males, alongside reproducing males in the population that defended territories near the old water source. By expanding the number of water sources, more adult bachelor males that did not have the opportunity to reproduce before, due to the lack of potential spaces near water sources, would be able to establish territories, thus gaining access to areas where they can secure matings with lactating females who drink daily and with non‐lactating females who drink more sporadically. This increase in the proportion of reproducing males among all adult males in the population should increase *N*_ev_, thereby enhancing the population's long‐term resilience by better preserving genetic diversity among foals in future generations (Greenbaum et al., 2018).

**FIGURE 1:**
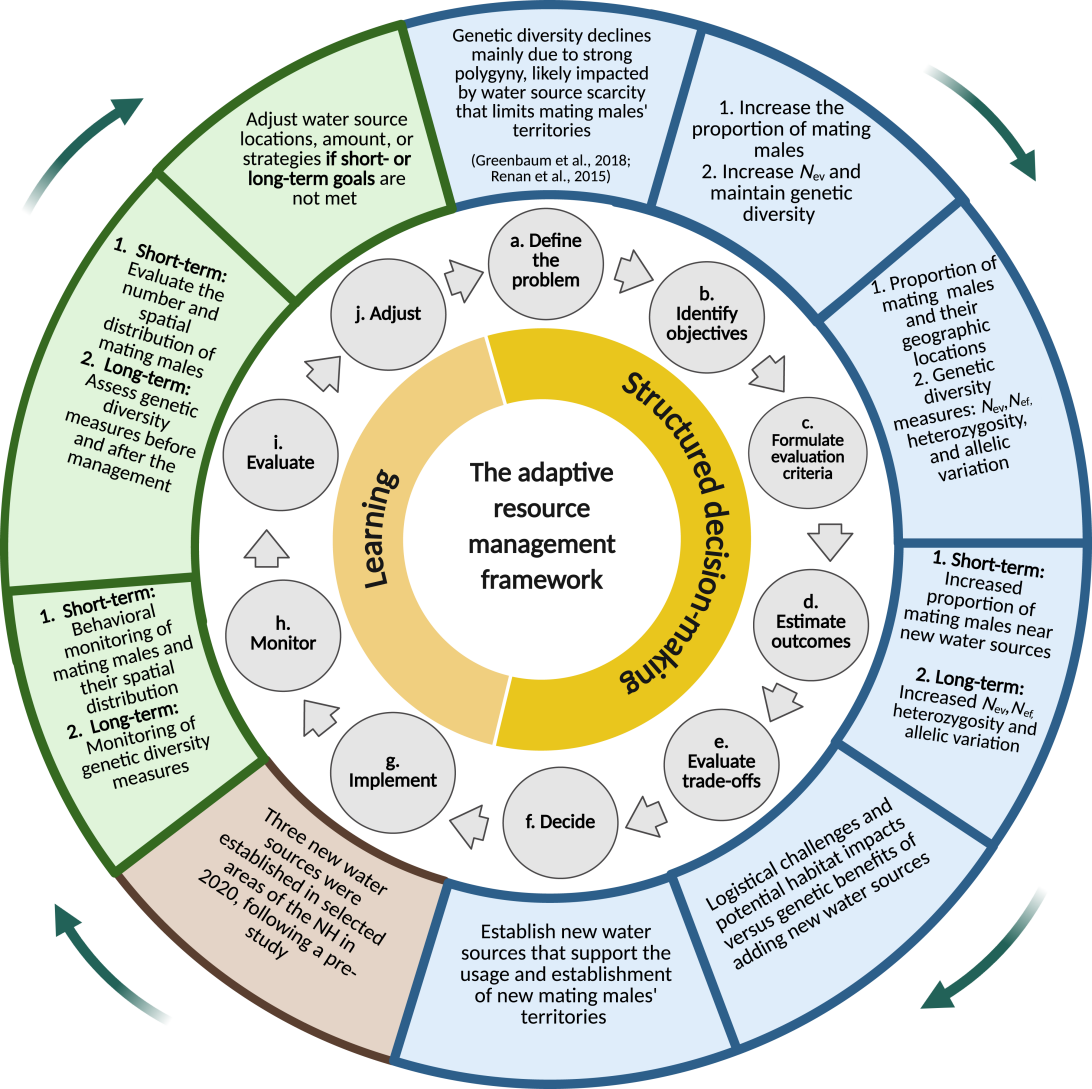
Overview of the adaptive management framework. Inner circle: the general basic model adapted by Allen et al. (2011); outer circle: the application to the Asiatic wild ass case study; see text (*Methods*) for further details. *N*_ef_, inbreeding effective size; *N*_ev_, variance effective population size; NH, Negev Highlands.

This water management was part of a comprehensive framework (Figure 1) based on the adaptive management approach (Lawler, 2009) in two main stages: short‐ and long‐term monitoring and evaluation. This approach recognizes that genetic effects occur over generations, thus requiring long‐term assessment with intermediate indicators to include behavioral response, which affects *N*_ev_. Specifically, we hypothesized that the behavioral response to the change in the water source distribution would occur quickly. We predicted that following the water source management, there would be (1) an immediate increase in the number of reproducing males out of all adult males in the population; (2) an increase in the number of reproducing males located near new water sources, potentially establishing territories for matings; and (3) an increase in *N*_ev_ as a result of the increase in the number of reproducing males.

## METHODS

### The resource management framework

We followed a model by Allen et al. (2011), originally developed for the conservation and management of natural resources in the adaptive management approach, adapting its basic stages to our specific case (Figure 1). “Adaptive management” is defined as “an iterative process where managers learn from experimental actions” (Lawler, 2009, p. 85). This approach involves monitoring outcomes and adjusting actions based on the system's needs (LeDee et al., 2021). Success is assessed by examining direct results or using proxy indicators for long‐term evaluation (McCarthy & Possingham, 2007). The following sections outline the main stages of the framework that can be applied to specific species and situations, and we present the Asiatic wild ass population case study as an example.

### Structured decision‐making: Problem definition and action planning

#### Study system

The Asiatic wild ass (*E. hemionus*) is an elusive, near‐threatened species found across central and eastern Asia and also historically in the Middle East (Kaczensky et al., 2025). Reintroduced into Israel in the early 1980s and 1990s (Saltz et al., 2000; Saltz & Rubenstein, 1995), *E. hemionus* now inhabits the Negev Desert (Zecherle et al., 2021). During the breeding season, activity is concentrated near water sources, mainly in the Negev Highlands (NH) (Renan et al., 2018). Gestation lasts 11 months, typically resulting in one foal, and females may give birth in consecutive years (Saltz & Rubenstein, 1995).

#### Problem definition

Due to limited undisturbed water sources in the region, the Israel Nature and Parks Authority (INPA) operate artificial water points in the Negev Desert (Nezer et al., 2017). These water points have been shown to influence the Asiatic wild ass's movement routes (Davidson et al., 2013), distribution (Nezer et al., 2017), habitat selection, and space‐use patterns (Giotto et al., 2015), as well as courtship and breeding (Renan et al., 2018; Saltz et al., 2000). Renan et al. (2015) found that fewer than 25% out of all adult males in the population reproduce and contribute to the gene pool. Greenbaum et al. (2018) identified that the proportion of reproducing males (10.6%–19.5%) was the primary factor leading to the low *N*_ev_. This issue was recognized as a conservation problem that should be addressed through active intervention (Figure 1; a) (Greenbaum et al., 2018; Renan et al., 2018).

#### Objectives, evaluation criteria, expected outcomes, and trade‐offs

Based on prior knowledge about the significant role of water sources in the mating system of the Asiatic wild ass population (see details above and in Rubenstein, 2010; Rubenstein et al., 2015; Saltz et al., 2000), they were selected as the focal point of the conservation action. The aim was to evaluate whether changing the distribution of water sources could increase the number of reproducing males out of all adult males in the population, which in turn would increase *N*_ev_ and promote genetic diversity over the long term. The management objectives (Figure 1; b), evaluation criteria (Figure 1; c), and expected outcomes (Figure 1; d) were as follows: (1) For the short term: (a) Objective: Increase the number of reproducing males by enabling more bachelor males to establish territories near newly accessible water sources (Greenbaum et al., 2018; Renan et al., 2018). (b) Evaluation criteria: The proportion of reproducing males in the population and their spatial distribution. (c) Expected outcome: A measurable increase in the number and proportion of reproducing males, reflected in a higher *N*_ev_ during the years following the intervention; (2) For the long term (i.e., a time period of at least one generation): (a) Objective: Reduce the rate of genetic diversity loss in the population by increasing the number of reproducing males that contribute to the population's gene pool over time. (b) Evaluation criteria: Genetic diversity measures such as *N*_ev_ and *N*_ef_ (based on molecular data, to distinguish current from future estimates), as well as allelic variation and heterozygosity (Allendorf et al., 2024). (c) Expected outcome: An increase in *N*_ev_ and *N*_ef_, indicating either improved maintenance of allelic diversity or a reduced risk of allelic loss across generations.

**FIGURE 2:**
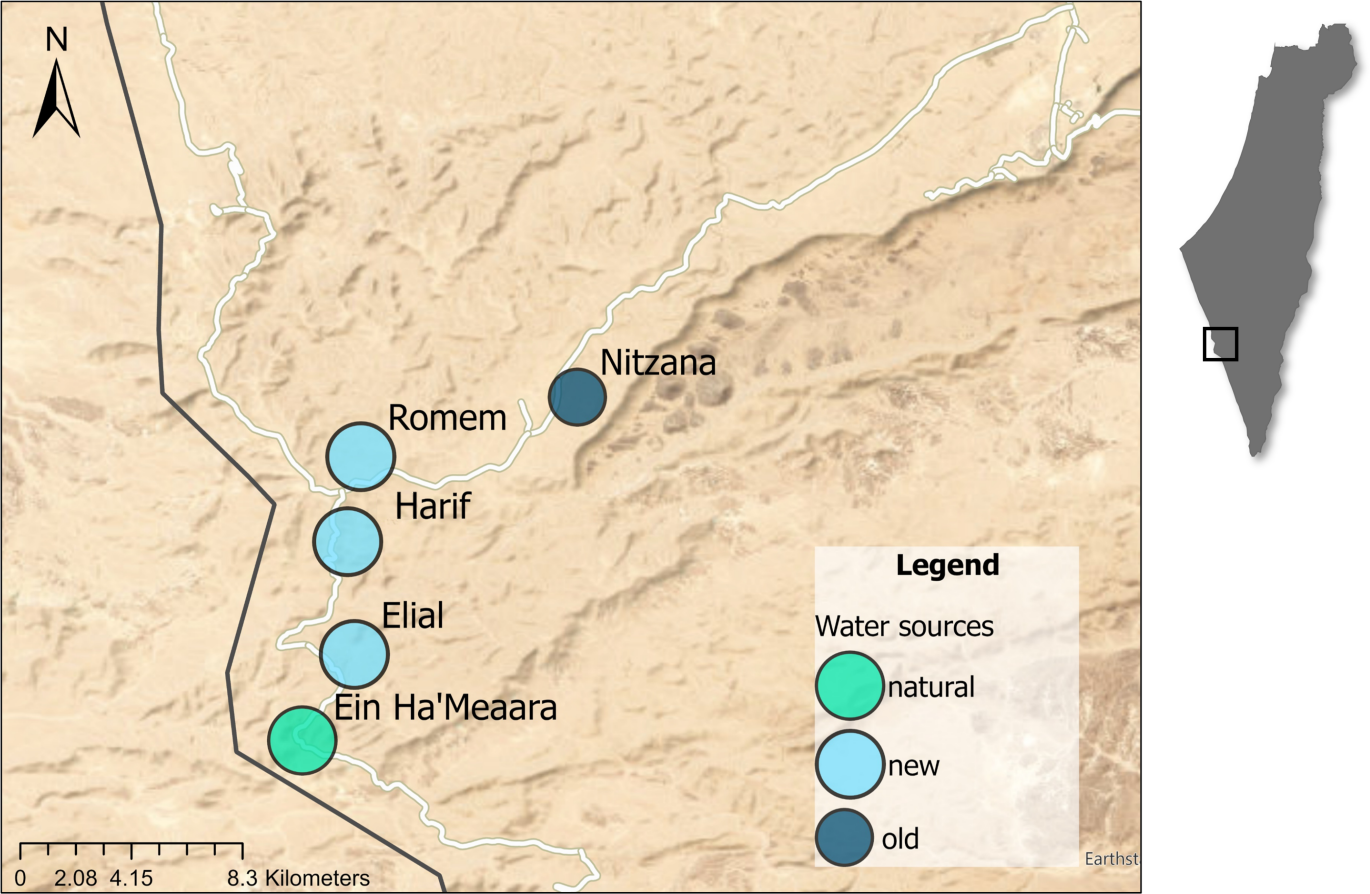
The artificial water sources in the Negev Highlands that support the Asiatic wild ass population during the breeding seasons. Nitzana: the old water source. Romem, Harif, and Elial: the new water sources. Ein Ha'Meaara: a natural spring that dried out in 2011, following a decrease in water levels, and was converted into an artificial water source.

As part of the trade‐off evaluation (Figure 1; e), we conducted a preliminary study to identify alternative water source sites. Biological factors (e.g., topography, plant cover, population connectivity) and logistical factors (e.g., distance from roads, proximity to water infrastructure) were considered (Appendix S1: Figures S1 and S2). Site selection was based on prior knowledge of space‐use and habitat preferences (Davidson et al., 2013; Giotto et al., 2015; Nezer et al., 2017). The potential benefits of providing additional space for reproducing males to establish territories (thereby increasing *N*_ev_) and reducing roadkill near the old water source, which was located close to the main road (Figure 2), outweighed the logistical challenges of establishing and maintaining water pipes. Thus, the decision was made to implement the water source intervention of shutting down the old water source and establishing three new artificial water sources to operate during the summer seasons, which is also the Asiatic wild ass breeding season (Figure 1; f).

### Learning: Implementation, monitoring, and evaluation

#### Implementation

The water source action was implemented in May 2020 (Figure 1; g). Three new water sources were established near the border between Israel and Egypt, located near the Romem Mountain, the Harif Mountain, and the Elial Wadi (Figure 2). These new water sources operated alongside the old source at the Nitzana Wadi (30.528987° N/34.644115° E) until the latter was closed, as planned, in September 2020 (Figure 2).

#### Monitoring

##### Sampling

For the short‐term monitoring stage (Figure 1; h), field surveys to collect dung for noninvasive genetic sampling and direct observations were conducted over two breeding seasons. To maximize foal captures, dung collection was carried out from 1 month after the first foals were born until 1 month after the breeding season ended (June–October). Sampling was conducted on 11 survey days in 2020 and 18 days in 2021. Multiple fecal samples were collected to identify unique genotypes and detect recaptures of adult females and males (i.e., females and males that were defined as adults based on dung size and are assumed to be sexually mature with reproductive potential) and foals (i.e., individuals that were born during the study period, as determined by dung size; see details below). This approach aimed to increase the probability of sampling reproducing males and their foals within the study area. Sampling followed the protocol of Renan et al. (2012) with slight modifications (Appendix S2). An ear swab dipped in inhibitEX buffer was used to collect surface fecal cells (Rutledge et al., 2010). Swabs were preserved in 1.5‐mL Eppendorf tubes, with 600 μL of inhibitEX buffer, and were kept in a portable cooler for several hours before reaching the lab.

Sampling occurred within 3–4 km of each water source and the main road (Figure 2) between 5:00 and 19:00 for 8–12 h per sampling day. Fresh droppings (i.e., feces whose outer and inner layers were still wet; Renan et al., 2012) were collected from directly and indirectly observed individuals. Age classification (i.e., adult or foal) was based on dung size (Kongrit & Siripunkaw, 2017)—a method that is more reliable when performed during the first several months after birth (April–September, Saltz & Rubenstein, 1995), when foal droppings are noticeably smaller than those of adults. For each sample, we recorded date, time, coordinates, age category (an adult or a foal, by dung size), sex (later determined by polymerase chain reaction [PCR]), and freshness (1–3 scale). For observed individuals, we recorded group details such as the number, sex, and age of individuals (Appendix S3: Table S1) (Kan‐Lingwood et al., 2024). Solitary males (i.e., adult males observed standing alone for a long period of time, showing aggressive or courtship behaviors, with the potential of being territorial, reproducing males) were photographed with a Nikon Coolpix P‐1000 camera. Individuals were socially categorized as solitary males, bachelor male groups, or adult females with young, based on Renan et al. (2018) (Appendix S3: Table S2). To avoid duplicate sampling, droppings within 1–2 m of each other were excluded (Gueta et al., 2014; Renan et al., 2015). Samples were stored at −20°C until DNA extraction. To ensure comparability across years and minimize methodological artifacts, we applied consistent sampling efforts, field protocols, and genotyping methods in both 2020 and 2021.

In addition to direct observations of males during fecal collection, the presence of solitary adult males near both old and new water sources (i.e., within a 3‐km radius) was assessed by direct observations of solitary males that were collected systematically between 2019 and 2021 along 39 predefined transects (Appendix S4: Figure S1). These observations provided complementary information to that gathered from genetic sampling on the locations and potential territories of reproducing males before, during, and after water source management.

##### DNA extraction, quantification, sex determination, and genotyping

DNA was extracted using the QIAamp FAST DNA Stool Mini Kit (Qiagen, Hilden, Germany) following the manufacturer's protocol with minor modifications (Appendix S5). Sex determination was performed using two sets of primers: eqZF9iR/eqZF9iF (Kim et al., 2009) and ESRY‐F002/ESRY‐R480 (Hasegawa et al., 2000). Each sample was amplified in three independent PCR reactions, with additional repeats for failures or mismatches (Kan‐Lingwood et al., 2024). AgriPlex Genomics (Cleveland, OH, USA) conducted DNA sequencing and genotyping via the PlexSeqTM platform. PCR primers were designed to amplify regions around each single nucleotide polymorphism (SNP). From thousands of detected SNPs previously designed from Asiatic wild ass blood samples of captive individuals in European zoos (Zecherle et al., 2021), we used only highly informative ones with a minor allele frequency (MAF) > 0.01 and at least 100 neighboring nucleotides, as required by AgriPlex Genomics and described by Kan‐Lingwood et al. (2024).

##### SNP and sample filtering

SNPs and samples were filtered following Kan‐Lingwood et al. (2024). Briefly, SNPs were filtered based on a threshold of at least 66% successful sequencing, and samples were filtered by having at least 70% sequencing success across SNPs. SNPs with an error rate above 1%, calculated using 255 (85 triplicate) samples, were also excluded. Recaptured individuals were identified by a minimum number of mismatching SNPs and separated into a dataset used later to test the year‐to‐year locations of reproducing males.

##### Genetic data analysis and parentage simulations

We calculated the probability of identity (PI) for our final genotype dataset using GenAlEx V 6.5 (Peakall & Smouse, 2012), which estimates the probability that two randomly drawn individuals share the same genotype across all loci (Peakall & Smouse, 2006). This is especially important when using noninvasive sampling and when there are no available data on relatedness between individuals in the population of concern (Ferreira et al., 2018; Taberlet & Luikart, 1999).

Given the importance of the number of genetic markers for accurate parental assignments (Foroughirad et al., 2019), we conducted simulations to ensure that the number of SNPs used provided sufficient resolution for reliable parentage detection (Kan‐Lingwood et al., 2026). The simulation followed these stages: (1) We created 100 simulated offspring (i.e., foals) using the R environment (R Core Team, 2023) from our resulting empirical dataset of unique genotypes (535 SNPs), with alleles inherited from randomly chosen adult females and adult males. Not all individuals were parents in the simulated offspring set due to the random sampling (62 mothers out of 90 adult females and 58 fathers out of 70 adult males). Some had more than one offspring (offspring per individual, mothers: mean: 1.6, max: 4; fathers: mean: 1.7, max: 5), reflecting the polygamy in the system. (2) We generated genotypes considering the known error rate per SNP (Kan‐Lingwood et al., 2024), replacing one allele with another if an “error” occurred. (3) We randomly chose different SNP sets from the 100 simulated offspring (535 SNPs) to create 10 different sets of offspring genotypes for 10 different sets of SNPs (50, 100, 150, 200, 250, 300, 350, 400, 450, 500 SNPs). This process created a total of 10 × 10 × 100 = 10,000 parentage tests (10,000 offspring). (4) To ensure the correct use of the final SNP set for parentage assignments (*n* = 535 SNPs), we ran the analysis for the final set 10 times with different random seeds, for a total of (100 × 10) + 10,000 = 11,000 parentage tests. (5) These genotypes (of both parents and simulated offspring) were input into COLONY v2.0.6.8 (Jones & Wang, 2010). The software parameters used were similar to those used for the empirical dataset (see below).

The identities of the resulting parents were then compared to the predefined identities of the simulated offspring's parents, and the number of correct assignments, as a function of the number of SNPs used in each parental test, was examined. We expected parental assignment accuracy to decrease with fewer SNPs (Foroughirad et al., 2019). This approach allowed us to check the consistency across runs and the convergence based on SNP number and to determine the minimum number of SNPs needed for reliable parentage assignments.

##### Parentage analyses

In order to assess the number of reproducing males in a given year during the research period, parentage assignments were conducted for foals born in the 2020 and 2021 breeding seasons. These included the identification of unique foals', adult males', and adult females' genotypes based on fecal samples using the filtering pipeline described in Kan‐Lingwood et al. (2024). After identifying unique genotypes and removing their resamples, we performed the parentage analysis. All adult males (including bachelor and reproducing males) and adult females sampled during the study were included as candidate sires and mothers, respectively. Reproducing males were defined as adult males assigned as fathers to foals based on the parentage analysis, including both those sampled directly and those inferred through pedigree reconstruction (see details below). We tested parental relationships in the empirical dataset using Cervus (Kalinowski et al., 2007) and COLONY programs to ensure result reliability, as shown in Wang and Santure (2009). Cervus uses a simulation approach to define a delta (∆) statistic based on population allele frequencies and resolves paternity between the two most likely adult males, assigning paternity to the adult male with the highest natural logarithm of the likelihood‐odds ratio (LOD) score if the difference from the second highest is larger than ∆ (Kalinowski et al., 2007; Marshall et al., 1998). We allowed for deviation from the expected 50% allelic match between parents and offspring. We used the exact value of the genotype error rate (*e* = 0.00174) based on its calculation in Kan‐Lingwood et al. (2024) as an input.

Unlike Cervus, COLONY infers parentage jointly, considering likelihood over the entire pedigree rather than individual pairs (Jones & Wang, 2010). The program uses maximum likelihood (ML) and pairwise likelihood approaches to assign and infer relationships (Jones & Wang, 2010; Wang, 2013a). For both programs, the software parameters used reflected the Asiatic wild ass mating system: female and male polygamy (Saltz et al., 2000), no inbreeding (Renan et al., 2015; Zecherle et al., 2021), and specific error rates per SNP (as calculated in Kan‐Lingwood et al., 2024). The “probability of candidate parents being present in the sample” parameter was tested separately across eight values (20%–90%). Of those, the value was chosen based on the highest assignment probabilities received in both Cervus and COLONY (for full details, see Appendix S6). Other parameters were set according to the default settings recommended by Jones and Wang (2010). In COLONY, we ran the test five times with different random seeds and high likelihood precision to ensure reproducibility and control the stochastic processes involved in the search for optimal parentage assignments. The pedigree output included parent–offspring relationships, and when parents were not present in the sample, the program inferred new genotypes for missing individuals, creating a more complete family pedigree (Wang, 2013b). Using this method allowed us to evaluate the number of reproducing males in each year, even if they were not directly sampled, providing an effective assessment of the changes in *N*_ev_ year‐to‐year based on demography (Greenbaum et al., 2018). Individuals were considered true parents only with confidence levels above 0.9 (for both ML and pairwise methods). Paternal assignments were then compared between the Cervus and COLONY programs.

#### Evaluation

This work presents the short‐term results of the resource management, focusing on the immediate changes in the proportion of reproducing males and their spatial distribution near the newly established water sources before and after the intervention (see Figure 1; i, short‐term monitoring). We also evaluated short‐term changes in genetic measures, including *N*_ev_, which was calculated based on direct demographic effects (sex ratio skew, eq. 1 in Greenbaum et al., 2018), and observed heterozygosity (*H*_o_) (see *Changes in N* _ev_ *and H* _o_ *year‐to‐year*), which was calculated from the genotypes of foals. Changes in *H*_o_ may reflect changes in mating patterns (e.g., García‐Navas et al., 2009).

##### Changes in the proportion of reproducing males

We analyzed year‐to‐year changes in the proportion of reproducing males out of all adult males in the population before and after water source intervention. This proportion was calculated by dividing the number of reproducing males (fathers inferred by Cervus and COLONY) by the estimated total number of adult males based on the “probability of candidate parents being present in the sample” parameter (detailed in *Parentage analyses*).

We tested the hypothesis that the number of single‐year reproducing males (i.e., reproducing males that sired foals in only one of the 2 years [2019 or 2020]) increased yearly. Given the high annual survival rate of males (Saltz & Rubenstein, 1995), we assume that most of the single‐year reproducing males were present in both years but reproduced in only one. The increase in single‐year reproducing males in 2020 was attributed to the addition of new water sources rather than to demographic factors (i.e., increase in the total number of adult males in the population), since survival is high, and annual changes in sexual maturation rates are not expected or, if present, are minor (Saltz & Rubenstein, 1995).

Using paternity and pedigree reconstruction from COLONY, we compared the proportion of reproducing males (fathers) from 2019 (premanagement) to 2020 (postmanagement). We included both sampled fathers and those inferred from the pedigree analysis (i.e., fathers not sampled but inferred from foal and female genotypes) (Jones & Wang, 2010). Reproducing males in 2019 were identified by their paternal assignments to the genotypes of foals born in 2020 and, in 2020, by paternal assignments to those of foals born in 2021.

The significance of differences was estimated using a two‐population proportions *z*‐test based on Freeman and Tukey's variance‐stabilizing transformation (Freeman & Tukey, 1950). The population proportion was defined based on Equation (1):

(1) $$\text{Population proportion}=\frac{\text{Total number of single}‐\text{year breeders}\;}{\text{Total number of breeding males of the same year}}.$$

The test uses an arcsin, square root transformation that stabilizes the sampling variances of proportions and allows for an easy adjustment of different sample sizes:

(2) $$a=\arcsin\sqrt{p_{i}},$$

where *p*_*i*_ is the proportion of interest in sample *i*, and the arcsin is measured using radians; the variance under the binomial sampling of sample size *N* for the proportion *p* is *pq*/*N*. In this case, the variance is a function of *p* and *N*, the latter of which is known, but *p* represents a “nuisance parameter.” However, the variance of *a* is given by 1/(4*N*), a function only of the sample size, simplifying the statistical analysis of proportions. When the sample size is small, this sample size is used to correct the transformation, where *n* is the sample size. When there are two samples, *i* and *j*, the test of the null hypothesis that *p*_*i*_ *= p*_*j*_ with the *z*‐statistic is

(3) $$z=\left(a_{i}-a_{j}\right)/\sqrt{1/\left(4n_{i}\right)+1/\left(4n_{j}\right)},$$

which is asymptotically distributed as a normal distribution (0,1) under the null hypothesis. The *z*‐statistic. therefore, tests the null hypothesis by using the standard normal distribution.

##### Changes in N_*ev*_ and H_*o*_ year‐to‐year

We calculated *N*_ev_ each year and compared the results between the years to evaluate whether short‐term changes in the number of reproducing males carry implications for long‐term changes in maintenance of genetic diversity (Kan‐Lingwood et al., 2026). Since our aim was to reduce the intensity of genetic drift through the number of individuals contributing to the variance of this small population's gene pool, *N*_ev_ was the most appropriate indicator to be used for this study (Ryman et al., 2019; Templeton, 2021; Waples, 2025). Because the foal sample sizes varied between years, we used a resampling approach adapted from Walker et al. (2020) to control unequal sample sizes. In each comparison, 10,000 random subsamples of foals were drawn from the larger dataset (foals born in 2021) to match the smaller sample size (foals born in 2020). For each iteration, *N*_ev_ was calculated using the demographic formula (Wright, 1931) for the effect of sex ratio skew, following Greenbaum et al. (2018):

(4) $$N_{\mathrm{ev}}=\frac{4N_{m}N_{f}}{\left(N_{m}+N_{f}\right)},$$

where *N*_m_ is the number of reproducing males and *N*_f_ is the number of reproducing females in the population during the given year. We used both sampled individuals and individuals inferred from the COLONY parentage assignments. The number of unique mothers and fathers was recorded from the parentage data for each resample. Only foals with both parents assigned were included in the *N*_ev_ calculation. The resulting distribution of *N*_ev_ values from the resampled dataset of the larger sample year was then compared to the fixed values from the smaller sample year. For a two‐tailed 5% significance level, an exact probability ≥0.975 indicated a significantly higher *N*_ev_ in the larger sample, while a probability ≤0.025 indicated a significantly lower *N*_ev_ (Walker et al., 2020).

We also calculated the observed heterozygosity, *H*_o_, based on foals' genotypes. Assessing *H*_o_ among offspring is another crucial indicator of the long‐term resilience of a population when targeting the maintenance of genetic diversity, especially in small populations (Zsolnai et al., 2025). We compared *H*_o_ between foals born in 2020 and foals born in 2021, using the formula

(5) $$H_{o}=\frac{\text{Number of heterozygous individuals}}{\text{Total number of individuals available}\;\mathrm{at}\;\text{the given locus}}.$$

Since the distributions of *H*_o_ across foals' genotypes had several outliers (see Appendix S7: Figure S1), we used a median test for the comparison using permutations. We first calculated the pooled median *H*_o_ across all individuals from both years. Individual foals were then classified as above or below this pooled median, creating a 2 × 2 contingency table with year and pooled median position as factors. We evaluated this contingency table using Fisher's exact test to determine whether there was a significant association between birth year and position relative to the pooled median. Additionally, we performed a Kolmogorov–Smirnov two‐sample test to compare the overall distribution shapes of *H*_o_ between the 2 years for a complementary assessment of differences in *H*_o_ distributions. All calculations of *H*_o_ and *N*_ev_ were performed in a customized script in the R environment (R Core Team, 2023).

##### Spatial distribution of reproducing males

We mapped the spatial sampling locations of reproducing males, including recapture locations, using ArcGIS 10.8.2 (ESRI Inc., 2018). We tested the hypothesis that the water management increased the presence of reproducing males (i.e., bachelor males that could not have established territories and reproduced before the intervention) near the new water sources, indicating the approximate location of their territories. Although not all reproducing males were observed during genetic sampling, feces locations provided insight into their space use. Reproducing males typically remain in their territories (estimated at approximately 1–4 km^2^, Giotto et al., 2015; Ziv, 2016) while occasionally leaving their territories to drink at the nearest water source (Renan et al., 2018; Saltz et al., 2000). We tested the increased presence near water sources by examining the geographic locations of reproducing males from 2019 (fathers of foals born in 2020) and 2020 (fathers of foals born in 2021), as inferred by COLONY and Cervus and sampled during 2020 and 2021.

To assess the presence of reproducing males near new water source during (2020) and after (2021) this management intervention, we tested whether the number of reproducing males increased within a 3‐km radius of each new water source in both of these years. Specifically, we tested whether adult males that did not reproduce in 2019 but did reproduce in 2020 were located near the new sources, implying the establishment of new territories in these areas. We analyzed this by (1) mapping the locations of the 2019 reproducing males in 2020 and 2021; (2) mapping the 2020 reproducing males during and after the water source management; (3) comparing the spatial locations of both the 2019 and the 2020 reproducing males during 2020 and 2021; and (4) highlighting new reproducing males in 2020 as sampled in 2020 and 2021. These results were interpreted in relation to the direct observation data of solitary males sampled between 2019 and 2021 (see details in *Sampling*).

## RESULTS

### Monitoring

#### Sampling, DNA extraction, PCR, and sequencing

A total of 864 samples were collected—296 in 2020 and 568 in 2021. Of these, 585 met the DNA criteria for sequencing—126 from 2020 and 459 from 2021. After removing four samples due to technical issues, 581 samples were sequenced—123 from 2020 and 458 from 2021. Including two replicates of 85 samples used as triplicates (i.e., an additional 170 samples) and five control blood samples from five different individuals (two males and three females) from the NH population, which were captured for GPS monitoring from 2012 to 2023 (described in Zecherle et al., 2021), the final sequencing set comprised 756 samples across 625 SNPs.

#### SNP and sample filtering

We excluded 51 SNPs and 49 samples based on sequencing success, which were lower than 66% and 70%, respectively. An additional 39 SNPs with an error rate ≥0.01 were removed, leaving 535 SNPs in the dataset (as described in Kan‐Lingwood et al., 2024). After filtration, the average error rate was 0.00174, with 92.6% sequencing success. The recapturing test identified 107 genotypes recaptured between 1 and 8 times each (Kan‐Lingwood et al., 2024). Specifically, among the 26 adult males recaptured, the number of recaptures per adult male ranged between 1 and 5 times. After excluding duplicates, the final dataset included 244 unique genotypes—90 adult females, 70 adult males, and 84 foals. Their locations were scattered across the study site (Appendix S8: Figure S1). The PI was 2.8E–119, far lower than the recommended 0.01 for a capture–recapture study (Taberlet & Luikart, 1999).

#### Parentage simulations

**FIGURE 3:**
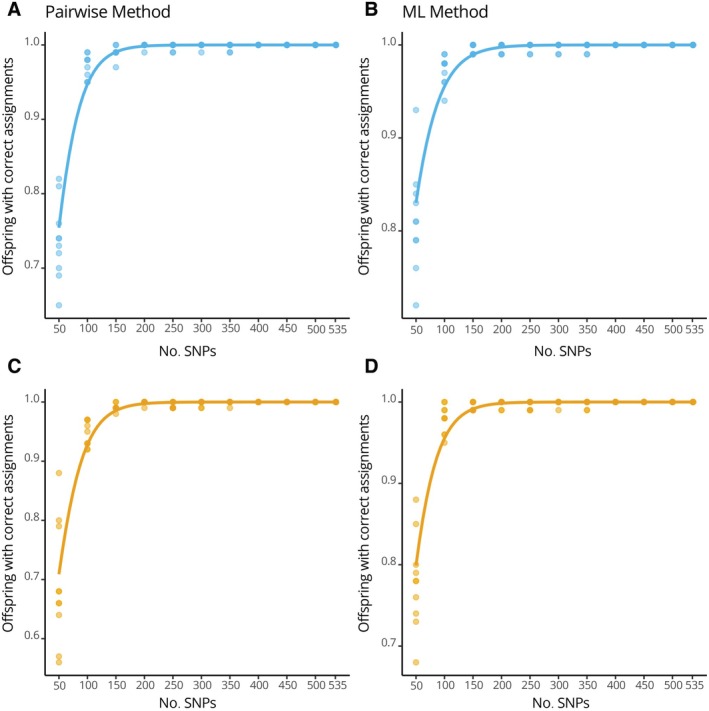
Parental simulation results. The graphs present the trend of correct paternal (A + B) and maternal (C + D) assignments as a function of the number of single nucleotide polymorphisms (SNPs) used in the analysis in the pairwise (A + C) and maximum likelihood (ML) (B + D) methods (1000 tests in total). Blue: paternity results; orange: maternity results. The line represents a logistic regression. Stable correct assignments are reached at about 400 SNPs.

The simulation test showed sufficient SNPs for a reliable parentage analysis: COLONY's pairwise and ML analysis showed complete and stable correct parentage assignments with 400 SNPs and above (Figure 3). Running 10,000 parental assignment replicates, 535 SNPs showed consistent trends and convergence in likelihood, paternity, and maternity assignments across iterations (Appendix S9 Figure S1).

#### Empirical parentage results

All parentage assignment tests were based on the identified 84 foals' unique genotypes (see *SNP and sample filtering*). Based on these assignments, the tests for estimating the “probability of candidate parents being present in the sample,” for the COLONY program settings, determined the value of 70%–80% as the most suitable for paternal assignments. This value indicates that the sampled adult males represent 70%–80% of the total number of adult males in the population. The parameter was set at 75% for all analyses (Appendix S6: Tables S1 and S2). Paternal assignment tests identified 20 matching fathers (sampled in the field) between programs: eight solitary males and two bachelors (as observed during their reproductive year) and 10 not directly observed. COLONY's pedigree reconstruction identified 27 additional reproducing males, totaling 47 across both years (Appendix S10: Tables S1 and S2). There were 40 matching paternal assignments with at least 95% confidence (Cervus) or 95% probability (COLONY) of sampled reproducing males. The number of foals per male ranged from 1 to 5. The total number of reproducing males, sampled and inferred by COLONY, was 16 in 2019 and 42 in 2020, with 11 reproducing males in both years (Table 1). As defined in *Empirical parentage results* section, these reproducing males, both those sampled and those inferred by COLONY pedigree reconstruction, are adult males identified as sires of foals' unique genotypes born during 2020 and 2021.

**TABLE 1:** Summary of paternity results.

Conception year	Sampled	Inferred by pedigree reconstruction	Total
2019	8	8	16
2020	17	25	42
Both years	5	6	11
Overall	20	27	47

*Note*: Number of reproducing males in 2019 and 2020 (i.e., year of conception) categorized by detection method (sampled or inferred by pedigree reconstruction).

### Evaluation

#### Changes in the proportion of reproducing males, *N*_ev_ and *H*_o_

Representing about 70%–80% of the population (see “probability of candidate parents being present in the sample” results in *Empirical parentage results* section), the 70 adult males sampled suggested there were approximately 88–100 adult males in the population, while the 90 adult females sampled suggested an estimated total of approximately 113–129 adult females. Based on the rationale that our sample represents 70%–80% of the population, we estimate 105–120 foals in 2020–2021 (84 sampled), which is consistent with equid reproductive biology where females usually produce one foal every 1–2 years (Rubenstein, 2011), although variation in reproductive success and foal survival makes this estimate less certain (Nunez et al., 2011).

Assuming no significant change in the total number of adult males between years, the estimated proportion of reproducing males out of all adult males in the population was 16%–18% in 2019 (16 out of 88–100, Table 1) and increased to 42%–48% in 2020 (42 out of 88–100, Table 1). This 2.6‐fold rise in reproducing males between 2019 and 2020 corresponds with the addition of three new water sources in the habitat, with the Harif water source being less used (see details below). Testing the change in the proportion of single‐year reproducing males out of all reproducing males in the population (i.e., those that reproduced only in either 2019 or 2020 but not in both), the *z*‐test showed a significant increase from 0.312 (5 out of 16) in 2019 to 0.738 (31 out of 42) in 2020 (*Z* = −2.877, *p* = 0.002, 95% CI for transformed proportions = [5.961, 7.113]).

The mean *N*_ev_ from the resampled smaller sample of 2021 was 38.4 (SD = 1.63), and 97.9% of the 10,000 simulation runs in this year had higher *N*_ev_ values than the observed *N*_ev_ of 2020 (*N*_ev_ = 34.9, see Appendix S7: Figure S2). This indicates a significant increase in *N*_ev_ in 2021 compared to 2020 (*p* = 0.979; recall from *Changes in N* _ev_ *and H* _o_ *year‐to‐year* section that the two‐tailed significance is defined as *p* < 0.025 for significantly lower *N*_ev_ and *p* > 0.975 for significantly higher *N*_ev_; Walker et al., 2020), even when controlling for sample size differences.

The median test showed no significant difference in *H*_o_ among foals born in 2020 and 2021. The pooled median *H*_o_ was 0.24, and exactly 50% of all foals from each year fell above and below this value (2020: 10 above, 10 below; 2021: 32 above, 32 below, see Appendix S7: Figure S1). Fisher's exact test showed no significant association between foals' birth year and whether individuals fell above or below the median *H*_o_ (*p* = 1), indicating that *H*_o_ did not differ between years. Similarly, the Kolmogorov–Smirnov test showed no significant difference between the *H*_o_ distributions of the 2 years (*D* = 0.23, *p* = 0.31). Although the 2020 foals had greater variability in *H*_o_ values (range: 0.22–0.45) compared to 2021 foals (range: 0.18–0.29) (Appendix S7: Figure S1), this difference in distribution was not significant.

#### Spatial locations of reproducing males

**FIGURE 4:**
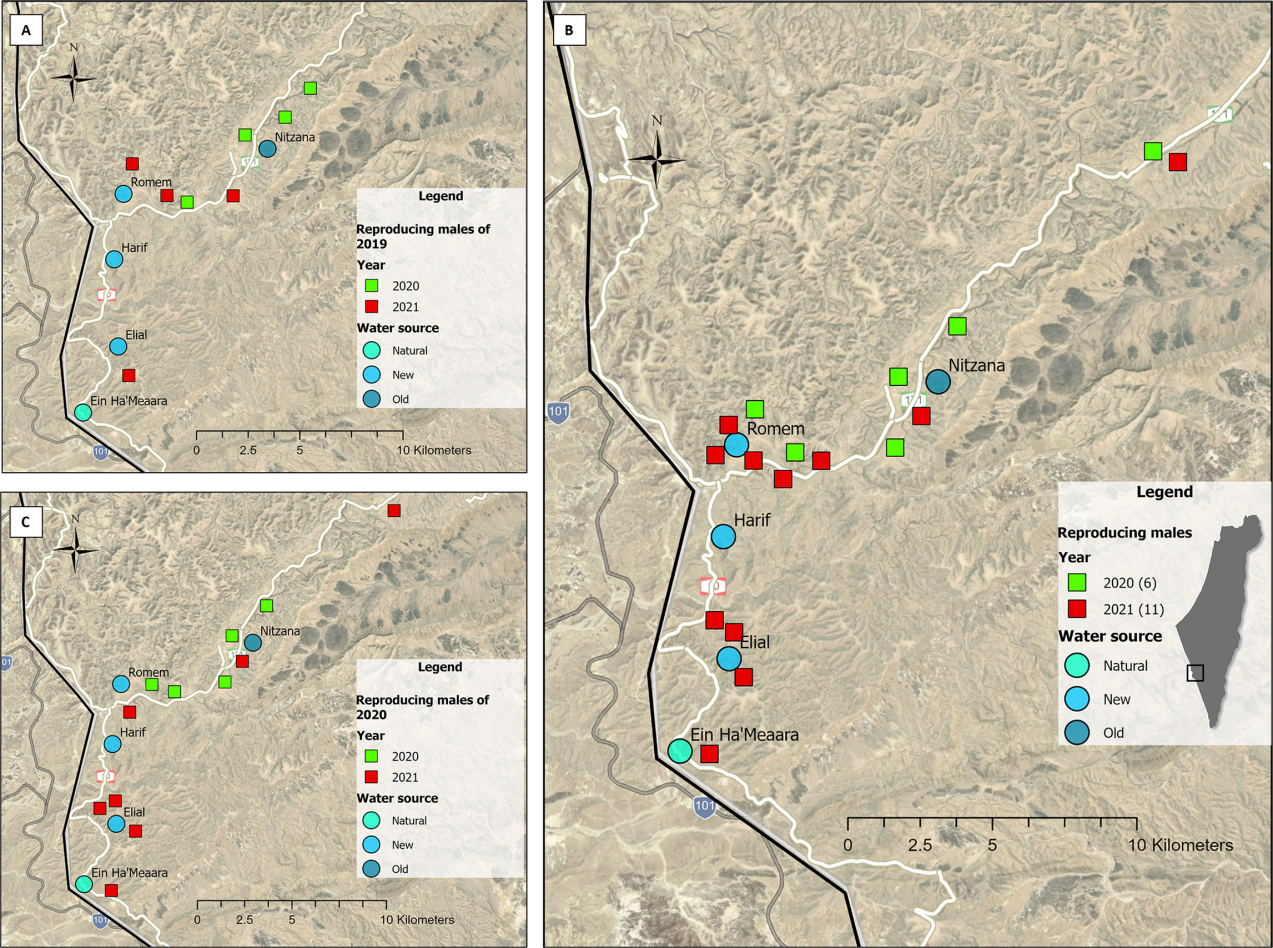
Geographical distribution of reproducing males by year and sampling period. (A) Eight reproducing males in 2019 (sires of foals born in 2020) that did not reproduce in 2020; four sampled in 2020 and four in 2021. (B) Spatial locations of 17 reproducing males in 2020 (sires of foals born in 2021), including six that were sampled in 2020 and 11 sampled in 2021; five of these males had also reproduced in 2019. (C) Spatial locations of 12 reproducing males in 2020 that did not sire foals in 2019. These are a subset of 31 sires of foals born in 2020 identified by parentage analysis; spatial data were unavailable for the remaining 19, identified only through pedigree reconstruction. Green squares represent reproducing males sampled in 2020; red squares represent reproducing males sampled in 2021. The figure demonstrates an increase in reproducing males' presence near the new water sources, particularly in 2021.

In accordance with the research hypothesis, an increase in the presence of reproducing males was observed near the new water sources in the year following the water source management (2021) compared to the year when the management took place (2020). Specifically, of the eight reproducing males in 2019 with foals born in 2020, four were sampled in 2020, and four were sampled in 2021 (Figure 4A). In 2020, when the old water source was still operating along with the three new water sources, three reproducing males were located near the old Nitzana water source, and one was located near the new Romem water source. In 2021, three reproducing males were located near the new Romem and Elial water sources, and one was located near the old water source.

Six of the 17 males that reproduced in 2020 were sampled during that same year, and 11 were sampled in 2021 (Figure 4B). In 2020, all six were found between the old Nitzana water source and the new Romem water source, with no reproducing males at the more distant new water sources. In 2021, 9 of the 11 males were found near the new water sources, including the distant ones (Harif and Elial), while only two were found near the old, dried‐up water source.

These results were further supported by direct field observations of solitary adult males collected from 2019 to 2021. Before 2020, solitary males were rarely observed near the new water sources (Appendix S4: Figure S2). During the management year, their presence in these areas increased, alongside a decrease within the 3‐km radius from the old Nitzana water source. In the year following the management (2021), their presence near the new water sources (Romem, Elial, and Harif) remained high, while fewer solitary males were observed near the old Nitzana water source.

To better understand the spatial positions of the new reproducing males, we examined the locations of 12 out of 31 identified reproducing males in 2020 that did not reproduce in 2019 and were directly sampled in the field (the other 19 out of 31 reproducing males were identified only through pedigree reconstruction based on parentage assignment [i.e., without direct sampling]; therefore, their spatial locations could not be determined [Table 1]). Among the 12 sampled reproducing males, five were sampled in 2020 and seven in 2021 (Figure 4C). All five reproducing males sampled in 2020 were found between the old Nitzana water source and the new Romem water source. In 2021, five reproducing males were found near the new water sources, including the distant ones, and two were found near the old Nitzana source.

Spatial recaptures of four males that reproduced in both 2019 and 2020 and recaptured across consecutive years (2020–2021) provided further support for shifts in the presence of reproducing males from the old to the new water sources (Appendix S8: Figure S2). Two reproducing males, ID 1511 and ID 1693, were recaptured at the same locations: male 1511 at the new Romem water source and male 1693 at the old Nitzana source. One male (ID 1533) was sampled yearly at the old Nitzana water source but was also sampled near Romem in 2021. Another reproducing male (ID 1595) was located at two different new water sources between the years—Romem in 2020 and Harif in 2021—indicating a spatial shift to the new water sources.

## DISCUSSION

This work provides empirical evidence that the number of breeding individuals and *N*_ev_ can be influenced by resource management in the short term. Specifically, we found that increasing the distribution of water sources increases the number and proportion of reproducing males out of all adult males in a population and their presence near new artificial water sources. In a resource defense polygyny mating system (Emlen & Oring, 1977), increasing the proportion of reproducing males is expected to increase the population's *N*_ev_—an important predictor of genetic diversity (Ryman et al., 2019; Waples, 2025). This intervention was performed as part of a management framework (Figure 1) that combines short‐term evaluation of reproduction responses (presented in this study) and long‐term estimation of genetic measures of the population, which requires further long‐term monitoring and evaluation. Our study demonstrates that resource intervention can influence behavior, reproduction, and short‐term genetic estimates in a population, which highlights the feasibility of targeting genetic measures and the importance of integrating genetic monitoring into adaptive management frameworks.

### Short‐term monitoring and evaluation of outcomes

Short‐term monitoring and evaluation are critical in assessing resource interventions' immediate impacts on the behavioral indicators of long‐term genetic effects. Our results support the theoretical prediction that, in territorial polygynous systems—where reproductive success is typically limited to a small subset of adult males that establish territories near water sources (Renan et al., 2015; Saltz et al., 2000)—expanding the number and distribution of water sources can enable more adult males that did not reproduce before (i.e., bachelor males) to establish territories and reproduce.

Paternity analysis revealed that the proportion of reproducing males among all adult males in the population was very low before management (16%–18% in 2019). This is consistent with Renan et al. (2015), who estimated that a range of 10%–25% of all adult males are reproducing males in the same population. After adding new water sources, the proportion of reproducing males increased to 42%–48%, with a significant rise in single‐year reproducing males in 2020 that had not sired foals in 2019 (*Z* = −2.877, *p* = 0.002). This 2.6‐fold increase in reproducing males aligns with the establishment of three new water sources and supports our prediction that increased water availability generates immediate behavioral change in mating patterns.

After controlling the sample size to account for potential sampling artifacts, *N*_ev_ in 2020 (38.4) was significantly higher than in 2019 (34.9; *p* = 0.979). This significant increase in *N*_ev_ indicates that the increase in reproducing males is expected to have a positive long‐term impact on the population's genetic diversity, with less allelic variation lost under genetic drift (Ryman et al., 2019; Templeton, 2021; Waples, 2025).

Unlike *N*_ev_, which is sensitive to short‐term demographic changes in the population, estimates of *H*_o_ among foals showed no significant year‐to‐year differences. This was expected at this short‐term evaluation stage, as meaningful changes in *H*_o_ typically emerge only after at least several generations following an event with a major impact on the population (Allendorf et al., 2024). This result further emphasizes the need for long‐term genetic monitoring within an adaptive resource management framework that includes the evaluation of long‐term resilience of the population using *H*_o_ among foals and other genetic estimators based on molecular data (e.g., *N*_ev_ based on the temporal method and *N*_f_ based on the linkage disequilibrium method; Zecherle et al., 2021; Zsolnai et al., 2025) to guide conservation goals.

The assumption that increasing the proportion of reproducing males in the Asiatic wild ass population of the Negev Desert would meaningfully impact genetic diversity relies on the genetic distinctiveness of individuals within the relatively small population. Although the population originated from a limited number of founders (Saltz & Rubenstein, 1995), its hybrid ancestry—derived from the Persian wild ass (*E. hemionus onager*) and the Turkmenian wild ass (*E. hemionus kulan*)*—*inflated its initial genetic diversity (Zecherle et al., 2021). Furthermore, Kan‐Lingwood et al. (2024), using the same dataset as the present study, found that unrelated individuals within this population differ at over 40% of the surveyed loci. This genetic difference means that increasing the number of reproducing males may help maintain the population's genetic diversity over time.

Spatial analyses further support the connection between resource distribution and mating structure. Here, the presence of new reproducing males (those that produced foals born in 2021) near the new water sources in 2020 and again in 2021 (Figure 4A–C; Appendix S8: Figure S2) aligns with our second prediction: that the presence of reproducing males would increase near the new water sources following the intervention. Recapture analysis revealed varied responses, with some reproducing males maintaining or shifting locations between years (Appendix S8: Figure S2). This result can be explained by changes in the spatial location of home ranges to cover new water sources or the establishment of new territories following the changing movement of adult females to the new drinking sites and potentially new vegetation surrounding them (Rubenstein, 1986). Also, the shift in reproducing males' locations can be explained as the maintenance of existing territories while moving to the new water sources for drinking. In our specific case, this might be explained by new water sources being established less than 10 km from the old water sources, surrounded by wadis with abundant forage. This is consistent with earlier findings by Saltz et al. (2000), who demonstrated that territorial reproducing males prefer areas near water sources and may hold territories for up to 7 years, provided they are near water sources where adult females drink and ideally near where adult females also prefer to eat (Rubenstein, 1986).

While some variation in sampling success between years may have occurred, consistent field protocols, identical analytical pipelines, and sample‐size corrections (when relevant) were applied across all years. Moreover, the spatial distribution of new reproducing males supports a behavioral pattern that is less likely to occur due to random chance: The majority of identified reproducing males that reproduced after the water source management and not before it were located near the newly established Romem, Elial, and (to a lesser extent) Harif water sources (Figure 4). Also, the concordance between the addition and relocation of water sources in the habitat and the emergence of new reproducing males—based on both genetic sampling (Figure 4) and direct observations (Appendix S4: Figure S2)— strengthen the interpretation that the change in reproducing males' presence near water sources reflects a behavioral response of adult males that could not have been territorial and reproduce before, rather than a sampling artifact.

Together, these results support the immediate influence of resource distribution on mating and space‐use patterns and support the first objective of the management framework: to increase the number of reproducing males out of all adult males in the population (see Figure 1; b). These findings align with other studies demonstrating how ecological interventions that alter resource distribution can rapidly shift mating behavior in wild populations (Cutting et al., 2021; Halliwell et al., 2016; Rubenstein, 2010).

### Alternative ecological explanations and study limitations

While our findings support a behavioral response to water source management, it is important to consider potential alternative explanations and study limitations. Some potential alternative ecological or demographic factors may have contributed to the observed pattern. One possibility is that the increase in reproducing males out of all adult males in the population between 2019 and 2020 could be partially influenced by demographic turnover—such as multiple males reaching sexual maturity simultaneously. However, even if a large number of adult males in 2020 had matured sexually, this alone is unlikely to explain the magnitude of the increase (from 16%–18% to 42%–48% reproducing males, i.e., a 2.6‐fold increase). In territorial polygynous systems such as that of the Asiatic wild ass, where only a small proportion of adult males establish territories and gain access to mates, not all sexually mature males become territorial or reproduce immediately after reaching sexual maturity (Rubenstein, 2010; Saltz et al., 2000).

Observational data from the NH population have shown that only a subset of adult males—those who secure territories—achieve reproductive success, while others remain nonreproductive and form nonstable bachelor groups (Saltz et al., 2000; Saltz & Rubenstein, 1995). In addition, territories are often stable, and dominant males may hold them for up to seven consecutive years (Saltz et al., 2000), suggesting that sexual maturity alone does not result in immediate reproductive participation. Although alternative male mating attempts such as sneaker strategies have been documented in some ungulates (Rubenstein, 1986), these are considered rare and unlikely to result in substantial paternity in the Asiatic wild ass population (Renan et al., 2015). Thus, the ability of adult males to become reproductive is constrained mainly by the possibility of establishing a territory in a suitable space near water or vegetation sources (Boyd et al., 2016).

Another possible explanation for the rise in reproducing males is that climatic variation—especially rainfall—may have affected habitat quality (e.g., forage and water availability), thus potentially facilitating the establishment of new territories near vegetation patches or temporary water sources. Saltz et al. (2006) found that higher precipitation in the year preceding conception, together with the absence of severe drought during gestation, increases foaling rates and early survival in the Asiatic wild ass population in the Negev Desert due to improved forage. Conversely, the establishment and maintenance of territories by Asiatic wild ass reproducing males are primarily constrained by access to constant water during the breeding season (Renan et al., 2018; Saltz et al., 2000), as water is essential for both females that drink daily while lactating (Nowzari et al., 2013) and reproducing males that defend territories surrounding water sources (Cao et al., 2025; Saltz et al., 2000; Saltz & Rubenstein, 1995). Therefore, while vegetation quality impacts female reproduction, foal survival, and territorial appeal, it is unlikely to fully explain the observed spatial increase in the proportion of reproducing males presented in this work.

We acknowledge that variation in sampling success across years could influence the identification of reproducing males, and we aimed to minimize this by using the same sampling and analysis pipeline each year. Since paternity was assigned based on foal genotypes rather than direct sampling of reproducing males, COLONY could infer fathers even if they were not sampled. By analyzing *N*_ev_ while correcting for differences in foal sample sizes (see *Changes in N* _ev_ *and H* _o_ *year‐to‐year*), we infer that the number of reproducing males increased year‐to‐year despite sampling variation. Also, the spatial overlap between new reproducing males and the new water sources (Romem, Elial, and Harif) (Figure 4; Appendix S4: Figure S2) supports a behavioral response to the intervention rather than a methodological artifact.

Finally, this study represents the first phase of a broader adaptive management framework (Figure 1) to monitor both short‐ and long‐term genetic outcomes. Our findings demonstrate immediate behavioral and spatial responses—consistent with the theory of resource defense polygyny—that are expected to precede changes in genetic diversity. The observed short‐term increase in reproducing males and *N*_ev_ provides initial empirical support for the effectiveness of the intervention. However, assessing long‐term genetic impact requires extended monitoring, especially in long‐lived species such as the Asiatic wild ass, which has a 7.5‐year generation time (Ransom et al., 2016). We, therefore, recommend integrating resource management into an adaptive framework that includes both short‐term (e.g., spatial use, reproductive success) and long‐term (e.g., *N*_ev_, *N*_f_, *H*_o_, *H*_*e*_, *N*_ev_, and *N*_f_ calculated based on genetic data; Zecherle et al., 2021) evaluations to guide future conservation actions.

### Long‐term monitoring and evaluation

Long‐term monitoring is essential to evaluate the actual impact of the management program on genetic diversity. Indicators that can be used to evaluate long‐term genetic effects include, for example, heterozygosity, allelic variation, and inbreeding coefficients over generations (Allendorf et al., 2024; DeWoody et al., 2021). Other measures can include those suggested here (Figure 1; d): *N*_ev_ and *N*_ef_ (Allendorf et al., 2024; Templeton, 2021; Zecherle et al., 2021), which are both directly linked to our short‐term behavioral indicators (Figure 1; d). For the Asiatic wild ass population in the NH, the short‐term increase in the number and proportion of reproducing males suggests a long‐term positive effect on *N*_ev_ and *N*_ef_ due to changes in the population's polygynous system (Greenbaum et al., 2018; Renan et al., 2015). These changes will be measured in future stages using the evaluation of *N*_ev_ and *N*_ef_ and heterozygosity (Figure 1; c,d) because they indicate long‐term changes in allelic variation caused by genetic drift and inbreeding between mating individuals (Greenbaum et al., 2018; Zecherle et al., 2021). Evaluating these estimators can also allow for a deeper understanding of such a management program's subsequent impact on evolutionary processes.

In addition to these genetic estimators, it is also important to continue monitoring behavioral and spatial responses. It is essential to monitor not only the defined long‐term genetic measures (Figure 1; d) but also the presence, behavior, and reproductive success of adult territorial males using direct observations combined with genetic sampling, as was done in the short‐term stage.

Effective long‐term monitoring requires setting clear objectives, considering the framework's short‐term findings (Allen et al., 2011). In cases where these are not achieved, adjustments should be considered to obtain the desired long‐term genetic outcomes. In the case of the Asiatic wild ass population, the short‐term findings aligned with our short‐term hypotheses (Figure 1), so no immediate adjustments were required. However, if reproducing males had not been sampled near the new water sources, adding water sources or relocating the water sources to more suitable areas, based on biological or technical criteria (Appendix S1), would have been necessary to obtain the desired behavioral responses.

### Practical application of the framework

Managing and monitoring genetic aspects of populations can be complex, particularly when aiming to detect processes shaping genetic diversity, following resource management (Ralls et al., 2018). Here, we suggest how managers can maximize the effectiveness of our framework by applying key principles at each step.

In the structured decision‐making phase (Allen et al., 2011, Figure 1), it is essential to first identify the resource with the greatest potential to impact the species' relevant behavioral responses (see *Problem definition* and the *Application to various systems* section below for further details on other resources with impacts on mating structure and genetic diversity). Second, a thorough assessment of logistical and biological considerations is necessary when selecting appropriate sites for resource distribution. This assessment should include an evaluation of physical and/or biological constraints in the habitat of concern, which may arise in future stages. For example, proximity to roads, agricultural fields, or hiking trails should be recognized as potential human–wildlife conflicts. At the same time, selecting areas with convenient access by car and on foot is important to enable rangers and researchers to conduct effective monitoring (see more examples in Appendix S1). Third, to determine expected genetic outcomes, the definition of clear intermediate targets in the short term should be emphasized to evaluate the management's long‐term success (Allen et al., 2011). This is particularly essential for long‐term genetic assessments, as it allows for adjustments when expected outcomes are not achieved (LeDee et al., 2021). For example, measurable indicators, such as individual presence near resources or changes in movement patterns, can guide adjustments to enhance gene flow and genetic connectivity.

In the learning phase (Allen et al., 2011; Figure 1), monitoring and evaluation methodologies should be selected to align with the program's aims to ensure that the approach meets the management's objectives. Here, for example, we used noninvasive genetic sampling and direct observations, which enabled us to perform various genetic analyses (e.g., individual identification and parentage analysis) and track individuals' geographical locations over time. This noninvasive methodological approach can also be relevant for other types of evaluation criteria, such as dispersal patterns and kinship (Ferreira et al., 2018), genetic censusing (Arandjelovic & Vigilant, 2018), and detection of critical mating areas (McFarlane et al., 2018), and it fits the “minimal disruption” strategy to evaluate potential genetic changes (Laikre et al., 2010; Ralls et al., 2018; Weeks et al., 2011).

### Application to various systems

Since the connection between resource distribution and sociality occurs across various species (Archie et al., 2008; Bowyer et al., 2020; Cotza et al., 2023; Marino et al., 2016), we suggest that our framework can be adapted to different species and systems in which low *N*_ev_ threatens genetic diversity. As natural water availability becomes limited in various ecosystems (Seddon et al., 2020), water management will be more critical across diverse habitats (Cantonati et al., 2020; Dai et al., 2018), and this work provides an example of how water sources can be effectively managed in such cases. Nevertheless, resources other than water can also impact movement and mating behavior. For instance, artificial food sources, such as feeding stations, impact movement patterns across different environments (Arkumarev et al., 2021; Penteriani et al., 2021; Thierry et al., 2020) and, therefore, carry the potential to be used as conservation tools to preserve genetic connectivity. Similarly, artificial shelters, such as roosts and nest boxes, provide breeding opportunities in habitats where natural resources are scarce (Crawford, 2024; Lee et al., 2023; Yu et al., 2022) and, therefore, may impact populations' *N*_ev_ by increasing mating encounters.

The case study of the Asiatic wild ass highlights a broader principle for conservation planning: In adaptive resource management, priority should be given to the resource expected to have the greatest influence on the desired behavioral change, thereby maximizing the effectiveness of the intervention. Also, our results show that even small‐scale, but planned, interventions—such as adding just a few resources in appropriate geographical locations—can achieve desired outcomes, including measurable impacts on behavior and reproduction. This approach can inform targeted management strategies in other systems, particularly where conservation resources are limited and focused actions are needed to increase the number of reproducing individuals in a population.

Beyond the impact on sociality and mating structure, resource distribution can impact the connectivity between subpopulations. By modifying movement patterns and space use, resource interventions can facilitate interactions between potential breeders from distinct subpopulations, thereby reducing inbreeding and genetic isolation through enhanced gene flow (Albert & García‐Navas, 2022; Burkhart et al., 2017). Therefore, the suggested framework can be particularly relevant for fragmented or genetically structured populations, where connectivity is critical for maintaining genetic diversity and adaptive potential (Chiu et al., 2023).

Our framework is particularly relevant when invasive approaches targeting genetic aspects (e.g., controlled breeding) are impossible or less favorable and a “bypassing” approach is more suitable. Weeks et al. (2011) suggested that indirect methods are often necessary to conserve genetic diversity, especially when there is a risk of outbreeding depression during genetic rescue or when reintroduction may cause captive adaptation. Hence, the framework suggested here aligns with conservation genetics principles, prioritizing maintaining evolutionary processes and genetic variability with minimum direct interference (Laikre et al., 2010; Ralls et al., 2018).

Overall, our findings from the Asiatic wild ass case study offer several overarching principles that can be used in other systems for other species. First, they demonstrate that targeted, short‐term interventions—such as adding only a few water sources—can substantially impact behavior, reproduction, and *N*_ev_, which is important for maintaining genetic diversity in the long term. This emphasizes the potential of resource‐based management as a noninvasive tool to reduce extreme reproductive skews in small populations, especially in systems where direct genetic interventions are impractical or harmful. Moreover, our results highlight the importance of defining and assessing short‐term objectives as part of an adaptive management process, since these are critical steps toward achieving long‐term conservation goals. Second, the study demonstrates that noninvasive methods for monitoring and evaluation constitute an efficient approach to assessing changes in genetic diversity in populations with elusive or wide‐ranging reproducing individuals. Finally, our framework emphasizes the importance of aligning conservation actions with the ecological and behavioral context of the species, particularly its mating system, to maintain genetic diversity. These conclusions are broadly relevant for species in fragmented landscapes or facing demographic constraints, where maintaining genetic diversity is essential for long‐term viability.

## AUTHOR CONTRIBUTIONS

Shirli Bar‐David, Amos Bouskila, Alan R. Templeton, and Daniel I. Rubenstein conceived the idea of this research. Shirli Bar‐David designed the genetic monitoring approach and integrated it into the adaptive management framework. Noa Yaffa Kan‐Lingwood conducted the fieldwork, collected the samples, and processed them with Naama Shahar. Amos Bouskila also formulated the adjustment of the framework for the adaptive management approach. Ariel Altman performed preliminary research on the establishment and management of water sources. Nurit Gordon contributed complementary information about spatial locations of solitary males from her fieldwork. Noa Yaffa Kan‐Lingwood and Alan R. Templeton designed the analyses. Liran Sagi designed the parentage simulations and, with Noa Yaffa Kan‐Lingwood, analyzed the results. Noa Yaffa Kan‐Lingwood wrote the original manuscript and revised it with Shirli Bar‐David. All authors commented on the manuscript.

## CONFLICT OF INTEREST STATEMENT

The authors declare no conflicts of interest.

## Supporting information


Appendix S1:

Appendix S2:

Appendix S3:

Appendix S4:

Appendix S5:

Appendix S6:

Appendix S7:

Appendix S8:

Appendix S9:

Appendix S10:

## Data Availability

Data and code to run the genotypes' filtration pipeline (Kan‐Lingwood et al., 2024) are available in Figshare at https://doi.org/10.6084/m9.figshare.24033723. Raw genotype data and the code to run parentage simulation and the *N*_ev_ and *H*_o_ analyses (Kan‐Lingwood et al., 2026) are available in Figshare at https://doi.org/10.6084/m9.figshare.28172192.
