# Supplementary material for: Resource management as a conservation tool to impact genetic diversity through mating patterns in wild populations
Source: Ecol Appl. 2026 Apr 2;36(3):e70226. doi: 10.1002/eap.70226 (PMC13044502; doi:10.1002/eap.70226)
Supplement: Supplementary file 1 — Appendix S1: [file EAP-36-e70226-s006.pdf]

Appendix S1

Title: Resource management as a conservation tool to impact genetic diversity through mating patterns in wild populations

Authors: Noa Yaffa Kan-Lingwood, Liran Sagi, Alan R. Templeton, Naama Shahar, Ariel Altman, Nurit Gordon, Daniel I. Rubenstein, Amos Bouskila, Shirli Bar-David

Journal: Ecological Applications

Artificial water source management planning phase

Background

This document summarizes the preliminary stage of water source management to study and recommend locations for new water sources in the Negev Highlands to support the Asiatic wild ass as the old water source in the Nitzana Wadi was shut down. Recommendations for the three new locations were based on GIS data analysis, discussions, and field tours, forming the basis for the management plan as applied by the Israel Nature and Parks Authority (INPA). The final decision regarding the locations was made by the INPA staff.

Prioritizing the optional locations

A total of 12 locations were initially assessed as potential placements for water sources in the Negev Highlands. Among them, three were dismissed early in the process due to camel presence, farmer activity nearby, the too great distance from the core distribution of the Asiatic wild ass population (Table S1), and their proximity to the existing water source in the Nitzana Wadi (“Nitzana”, Figure S1). The nine sites that were further evaluated are presented in Figure S1. Ein Ha'Meaara (Figure S1) was eventually dismissed from the options because of low Asiatic wild ass activity in its surrounding area (see habitat suitability and highly active areas in the Negev Highlands, Figure S2A, S2C, S2D). An additional location near the Bator Valley was also found unsuitable due to differences in elevation, geology, and vegetation, and the distance from the existing population in the Negev Highlands. Hence, the prioritization process focused on seven locations north of the Lutz Cliffs line (Figure S1, Table S1).

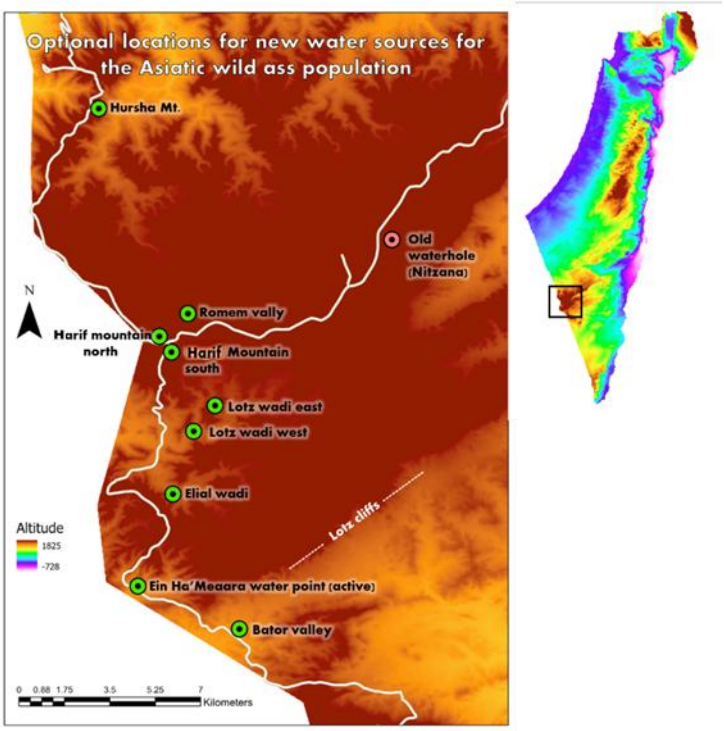

Figure S1. DEM of the Negev Highlands and the nine locations that were checked as potential areas to establish new water sources for the Asiatic wild ass population.

To prioritize and select the three most suitable locations from the seven remaining options (Hursha Mountain, Romem Valley, Harif Mountain (north and south), Lotz Wadi (east and west), and Elial Wadi; Table S1), a list of 15 criteria were developed based on known needs from interface and research. Each location was assessed using a score from 0 ('Not suitable') to 2 ('Suitable') for each criterion. These scores were based on accumulated knowledge of the Asiatic wild ass population's ecology, behavior, and genetics (Giotto et al., 2015; Nezer et al., 2017; Renan et al., 2015), along with expert input and ArcGIS 10.5 data analysis (examples in Figure S2). After evaluating all criteria, each location was assigned an overall rating (Table S1).

Table S1. Evaluation criteria for the suitability of locations for the new water source establishment (scores range from 0–2; 0: low suitability, 2: high suitability).

	Criterion	Water sources' locations (see Figure S1)						
		Hursha Mountain	Romem Valley	Harif Mountain North	Harif Mountain South	Lotz Wadi East	Lotz Wadi West	Elial
	Habitat-related factors							
1	Access for the Asiatic wild asses	0.5	2	1	0.75	2	1.9	2
2	Distance from roads	0.114	0.57	0.143	0.03	2	1.6	0.74
3	Landscape connectivity from the old water source to the focal one	0	1.5	1	0.9	2	1.1	1.25
4	Proximity to the old water source (Nitzana)	1.8	1.1	1.4	1.4	1.4	1.6	2
5	Connectivity to other populations	0	0	0	0	0	0	1
6	Proximity to the Egyptian border and fire zones	0	1	0	0	1	1	0
7	Habitat suitability (following Nezer et al., 2017)	0.75	0.75	0.75	0.5	1	1	1

8	Plant cover (following Nezer et al., 2017)	2	1.878	1.681	1.519	1.425	1.327	1.257
9	Topographical similarity to the old water source	1	2	1.5	1	1.5	1.25	1.5
10	The potential to support the wild ass population in the central Negev Highlands	0	1	1	1	1.5	1.5	2
	Factors related to external parties							
11	Access for travelers	0.5	0	0.5	0.5	0	0	0.5
12	Camel activity	1.5	1	1.25	1.25	1.5	1.5	2
13	Distance from agricultural lands	0.5	1	1	1	1.5	1.5	2
	Logistical aspects for research and maintenance							
14	Access by car and on foot to the water source and its surroundings by rangers and researchers	0	1	0.5	0.5	1.5	2	0.5
15	Distance from local water infrastructure to which pipes can be attached	0	1	1.5	1.5	0.5	1	2
	Total	8.7	15.8	13.2	11.8	**18.8**	**18.3**	**19.7**

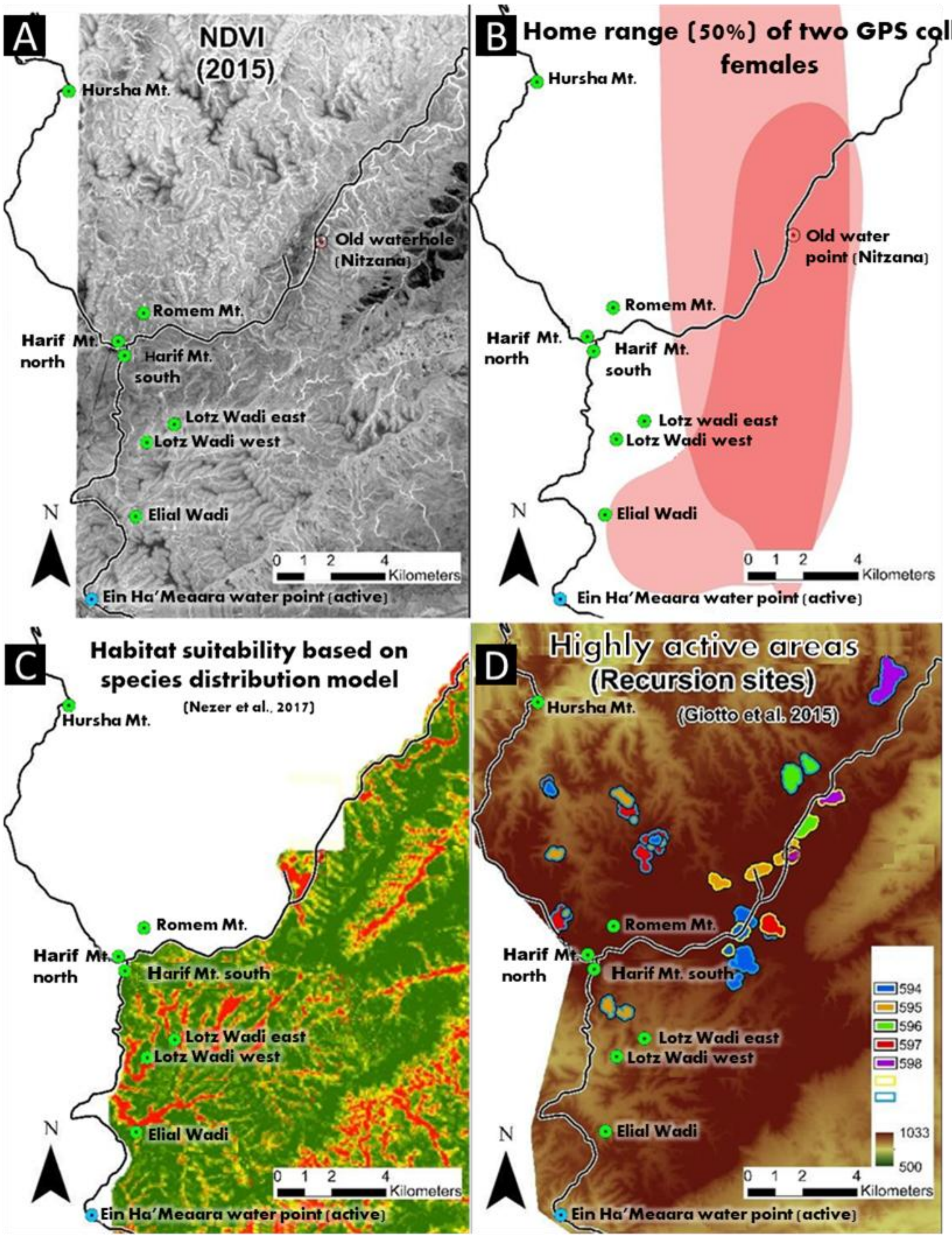

Figure S2. Examples of GIS analyses used as the basis for location scoring are given in Table S1.A: NDVI in the Negev Highlands. B: Home ranges of two GPS-collared females. C: Habitat suitability of the Asiatic wild ass based on distribution model (Nezer et al., 2017); D: Recursion sites of five GPS-collared individuals (Giotto et al., 2015).

Recommendations and summary

Elial Wadi, Lotz Wadi East, and Lotz Wadi West were the three locations with the highest scores (Table 1). However, due to the research advantage of scattering the water sources across a large area, with varying distances from the old water source in the Nitzana Wadi, the recommended locations were the

Elial Wadi, the Lotz Wadi (east or west), and the Romem Valley. Ultimately, logistical factors (presented by the INPA rangers) led to the final choice made by the INPA staff of two out of these recommended sites, the Elial Wadi and the Romem Valley (Figure S2), and the selection of Harif Mountain South as the third site (despite its relatively lower rank (Table 1).

References

Giotto, N., Gerard, J. F., Ziv, A., Bouskila, A., Bar-David, S. (2015). Space-use patterns of the Asiatic Wild Ass (*Equus hemionus*): Complementary insights from displacement, recursion movement, and habitat selection analyses. *PLoS ONE 10*(12), e0143279. https://doi.org/10.1371/journal.pone.0143279

Nezer, O., Bar-David, S., Gueta, T., Carmel, Y. (2017). High-resolution species-distribution model based on systematic sampling and indirect observations. *Biodiversity Conservation, 26*, 421–437. https://doi.org/10.1007/s10531-016-1251-2

Renan, S., Greenbaum, G., Shahar, N., Templeton, A. R., Bouskila, A., Bar-David, S. (2015). Stochastic modeling of shifts in allele frequencies reveals a strongly polygynous mating system in the re-introduced Asiatic wild ass. *Molecular Ecology, 24*, 1433–1446. https://doi.org/10.1111/mec.13131
