# Supplementary material for: Resource management as a conservation tool to impact genetic diversity through mating patterns in wild populations
Source: Ecol Appl. 2026 Apr 2;36(3):e70226. doi: 10.1002/eap.70226 (PMC13044502; doi:10.1002/eap.70226)
Supplement: Supplementary file 2 — Appendix S2: [file EAP-36-e70226-s007.pdf]

Appendix S2

Title: Resource management as a conservation tool to impact genetic diversity through mating patterns in wild populations

Authors: Noa Yaffa Kan-Lingwood, Liran Sagi, Alan R. Templeton, Naama Shahar, Ariel Altman, Nurit Gordon, Daniel I. Rubenstein, Amos Bouskila, Shirli Bar-David

Journal: Ecological Applications

Collection of fecal samples using a swab

What to prepare?

Gloves with talc

1.5-ml Eppendorf tubes numbered and filled with 600 μl inhibitEX buffer

Plastic Q-tips (swabs) cut in half (so the tube can be closed with the tip inside)

How to collect?

- Moisten the Q-tip by dipping it in the inhibitEX buffer (in the tube)
- Gently wipe the outer layer of one feces with the swab (moisten again between feces)
- Put the Q-tip in the tube with the swab facing down and close it
- Record the location, date, status of the feces, and individual sex and age (if known)

Preservation

Place the tubes in the fridge at 4°C; DNA can be extracted even after several months.
