# Supplementary material for: Resource management as a conservation tool to impact genetic diversity through mating patterns in wild populations
Source: Ecol Appl. 2026 Apr 2;36(3):e70226. doi: 10.1002/eap.70226 (PMC13044502; doi:10.1002/eap.70226)
Supplement: Supplementary file 3 — Appendix S3: [file EAP-36-e70226-s002.pdf]

Appendix S3

Title: Resource management as a conservation tool to impact genetic diversity through mating patterns in wild populations

Authors: Noa Yaffa Kan-Lingwood, Liran Sagi, Alan R. Templeton, Naama Shahar, Ariel Altman, Nurit Gordon, Daniel I. Rubenstein, Amos Bouskila, Shirli Bar-David

Journal: Ecological Applications

Field fecal sample collection method

Table S1. Fecal sample collection format.

ID	Date	Hour	Loc_E	Loc_N	Sex	Age	Fresh (1-3)	Coll_By	Dung Pile (Yes/No)	Pic_No	Direct Obs (Yes/No)	Comments

Fresh: 1: wet outside, 2: dry outside but wet inside, 3: dry outside and inside.

Comments: collection area, group size, other.

Table S2. Social categorization of directly observed individuals when sampled (based on Renan et al., 2018).

Social category	Description
Solitary male	An adult male observed to be solitary while walking, foraging, roaming, standing, showing dominant behavior, harassing female groups, and/or aggressively fighting with another male/s, and who does not belong to a particular bachelor group.
Bachelor male	An adult male observed in a group of at least one or more adult males.
Adult females with their young	An adult female observed alone or with a foal, as well as a groups of females with foals/yearlings. Only infrequently accompanied by one or two males.

References

Renan, S., E. Speyer, T. Ben-Nun, A. Ziv, G. Greenbaum, A. Templeton, S. Bar-David, and A. Bouskila. 2018. "Fission-fusion Social Structure of a Re-introduced Ungulate: Implications for Conservation." *Biological Conservation* 222: 261-267. https://doi.org/10.1016/j.biocon.2018.04.013.
