# Supplementary material for: Resource management as a conservation tool to impact genetic diversity through mating patterns in wild populations
Source: Ecol Appl. 2026 Apr 2;36(3):e70226. doi: 10.1002/eap.70226 (PMC13044502; doi:10.1002/eap.70226)
Supplement: Supplementary file 4 — Appendix S4: [file EAP-36-e70226-s005.pdf]

Appendix S4

Title: Resource management as a conservation tool to impact genetic diversity through mating patterns in wild populations

Authors: Noa Yaffa Kan-Lingwood, Liran Sagi, Alan R. Templeton, Naama Shahar, Ariel Altman, Nurit Gordon, Daniel I. Rubenstein, Amos Bouskila, Shirli Bar-David

Journal: Ecological Applications

Changes in solitary males' presence near the old and new water sources from 2019–2021 based on direct observations

Direct observations were used to assess whether spatial shifts in solitary males—which are the reproducing males in the Asiatic wild ass (*Equus hemionus*) mating system (Boyd et al., 2016; Renan et al., 2018; Saltz & Rubenstein, 1995; Saltz et al., 2000)—could be attributed to the water source management intervention. Field surveys were conducted between 2019 and 2021. Observations were carried out once a week during daylight hours along 39 fixed transects (Figure S1), using car and foot patrols, and from lookouts overlooking artificial water sources. Each field day included a different set of transects, visited at varying hours. At least one water source was visited per day, where observations were conducted from a distant vantage point for a minimum of 30 minutes.

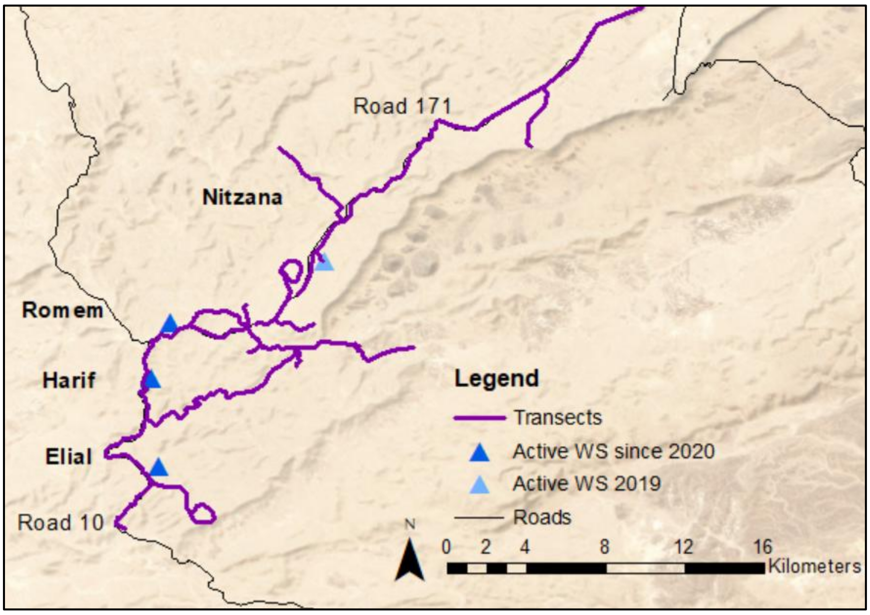

Figure S1. Transects in the study area of the Negev Highlands (marked in purple).

The start time of each transect was recorded. When wild asses were observed, their location, age, and sex class (adult female, adult male, or foal) were documented using the SW Map application on a mobile phone, along with the time of observation and the transect from which they were sighted. For water source observations, both the start and end times were recorded. Additionally, we recorded whether each observed individual was already present at the water source at the beginning of the observation or if it arrived during the observation period. Individual identification and characterization

were conducted using Nikon Monarch 5 M511 10x42 binoculars and a Nikon COOLPIX P1000 camera.

Results

A noticeable shift in the spatial distribution of solitary wild asses occurred over the period before, during, and after the water source management. In 2019, before the intervention, most solitary males were observed around the Nitzana (old) water source (Figure S2). In the subsequent years (2020–2021), solitary males were increasingly observed near the newly activated water sources, Romem, Charif, and Elial, which were established in 2020.

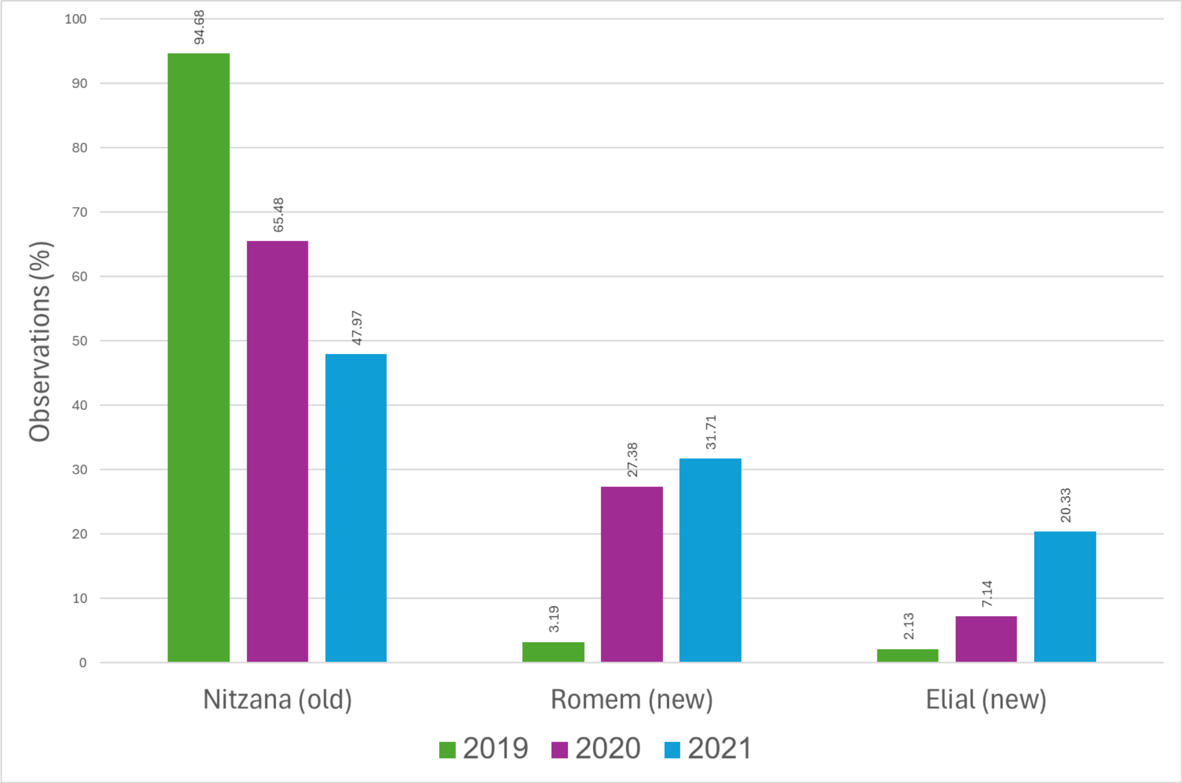

Figure S2. Observations near the old water source at Nitzana gradually declined from 94.68% in 2019 (the highest recorded value) to 47.97% in 2021 (the lowest). In contrast, observations near the new water sources—Romem and Elial (which also includes the surrounding 3-km radius of Harif)—increased steadily over the same period.

References

Boyd, L., A. Scorolli, H. Nowzari, and A. Bouskila. 2016. "Social Organization of Wild Equids." In *Wild Equids: Ecology, Management, and Conservation*, edited by Jason I. Ransom and Petra Kaczensky, 7-22. Johns Hopkins University Press.

Renan, S., E. Speyer, T. Ben-Nun, A. Ziv, G. Greenbaum, A. Templeton, S. Bar-David, and A. Bouskila. 2018. "Fission-fusion Social Structure of a Re-introduced Ungulate: Implications for Conservation." *Biological Conservation* 222: 261-267. https://doi.org/10.1016/j.biocon.2018.04.013.

Saltz, D., and D. I. Rubenstein. 1995. "Population Dynamics of a Re-introduced Asiatic Wild Ass (Equus hemionus) Herd." *Ecological Applications* 5: 327-335. https://doi.org/10.2307/1942025.

Saltz, D., M. Rowen, and D. I. Rubenstein. 2000. "The Effect of Space Use Patterns of Reintroduced Asiatic Wild Ass on Effective Population Size." *Conservation Biology* 14: 1852. https://www.jstor.org/stable/2641537.
