# Supplementary material for: Resource management as a conservation tool to impact genetic diversity through mating patterns in wild populations
Source: Ecol Appl. 2026 Apr 2;36(3):e70226. doi: 10.1002/eap.70226 (PMC13044502; doi:10.1002/eap.70226)
Supplement: Supplementary file 5 — Appendix S5: [file EAP-36-e70226-s010.pdf]

Appendix S5

Title: Resource management as a conservation tool to impact genetic diversity through mating patterns in wild populations

Authors: Noa Yaffa Kan-Lingwood, Liran Sagi, Alan R. Templeton, Naama Shahar, Ariel Altman, Nurit Gordon, Daniel I. Rubenstein, Amos Bouskila, Shirli Bar-David

Journal: Ecological Applications

DNA extraction using QIAamp Fast DNA Stool Mini Kit

Equipment needed:

1. QIAamp Fast DNA Stool Mini Kit
2. Microcentrifuge (with rotor for 2-ml tubes)
3. Heating block
4. Ethanol (96–100%)
5. 1.5-ml microcentrifuge tubes
6. 2-ml microcentrifuge tubes
7. Pipette tips with an aerosol barrier
8. Disposable tweezers
9. Biohazard can
10. Bleach can

Before starting:

1. If a precipitate has formed in the InhibitEx buffer or AL, dissolve by incubating at 70°C until fully dissolved.
2. Ensure that ethanol has been added to Buffer AW1 and Buffer AW2.
3. Mix all buffers before use.
4. Heat the block to 70°C for use in step 2.
5. Heat the ATE buffer (200 uL × sample number) to 56°C for steps 18–19.
6. Turn the swab upside down in the tube so that the side used for wiping is face up.

Replace tweezers and gloves between samples and try not to wet the tube lid.

Put tweezers in bleach.

7. Centrifuge the tubes (13,300 rpm) for 1 min.

so that all the supernatants emerge from the absorbent end of the swab.

8. Remove the swab with tweezers and throw it into the biohazard can.

Leave the supernatant in the tube.

9. Pipette 20 µl Proteinase K into a new 2-ml microcentrifuge tube (not provided).

10. Pipette all supernatant from step 3 (~400 µl) into the 2-ml microcentrifuge tube containing proteinase K.

11. Add 600 µl of AL buffe and vortex for 15 sec.

It is essential that the sample and the AL buffer are thoroughly mixed to form a homogeneous solution.

12. Incubate at 70°C for 10 min.

Centrifuge briefly to remove drops from the inside of the tube lid.

13. Add 600 µl ethanol (96–100%) to the lysate and mix by vortexing.

Centrifuge briefly to remove drops from the inside of the tube lid.

14. Carefully apply 600 µl lysate from step 10 to the QIAamp Spin Column (in a 2-ml collection tube) without wetting the rim. Close the cap and centrifuge at full speed for 1 min. *If the lysate has not entirely passed through the column after centrifugation, centrifuge again until the QIAamp spin column is empty.*

15. Place the QIAamp Spin Column in a clean 2-ml collection tube (provided) and discard the tube containing the filtrate**.**
16. Repeat steps 11–12 until all of the lysate has been loaded on the column.
17. Carefully open the QIAamp Spin Column and add 500 µl Buffer AW1 without wetting the rim. Close the cap and centrifuge at full speed for 1 min.
18. Place the QIAamp Spin Column in a clean 2-ml collection tube (provided) and discard the collection tube containing the filtrate.
19. Carefully open the QIAamp Spin Column and add 500 µl Buffer AW2 without wetting the rim. Close the cap and centrifuge at full speed for 3 min.

Note: Residual Buffer AW2 in the eluate may cause problems in downstream applications.

20. Discard liquid from the collection tube and replace the Spin Column in the collection tube.
21. Centrifuge again at full speed for 3 min.

This step helps to eliminate the possibility of Buffer AW2 carryover.

22. Transfer the QIAamp Spin Column to a new labeled 1.5-ml microcentrifuge tube (not provided) and discard the collection tube containing the filtrate.
23. Carefully open the QIAamp Spin Column and add 100 µl (prewarmed) of the ATE buffer directly onto the QIAamp membrane. Incubate at room temperature (15–25°C) for 5 min, then centrifuge at 8000 rpm for 1 min to elute DNA.
24. Repeat step 20.

The DNA is now in the ATE buffer and ready for use**.**
