# Supplementary material for: Resource management as a conservation tool to impact genetic diversity through mating patterns in wild populations
Source: Ecol Appl. 2026 Apr 2;36(3):e70226. doi: 10.1002/eap.70226 (PMC13044502; doi:10.1002/eap.70226)
Supplement: Supplementary file 6 — Appendix S6: [file EAP-36-e70226-s003.pdf]

Appendix S6

Title: Resource management as a conservation tool to impact genetic diversity through mating patterns in wild populations

Authors: Noa Yaffa Kan-Lingwood, Liran Sagi, Alan R. Templeton, Naama Shahar, Ariel Altman, Nurit Gordon, Daniel I. Rubenstein, Amos Bouskila, Shirli Bar-David

Journal: Ecological Applications

Testing the 'probability of candidate parents being present in the sample' parameter for parentage analyses in Cervus and COLONY

To define the parameter required in both Cervus (termed 'proportion of candidate parents sampled'; Marshall et al., 1998) and COLONY (termed 'probability of true parents being sampled'; Jones & Wang, 2010), a series of tests were conducted using paternity analysis. In each test, only the specific parameter value was systematically changed on a scale from 0.2 to 0.9 (i.e., a minimum probability of 20% to a maximum of 90%) in increments of 0.1 between each run for eight tests. Each test was repeated five times to ensure consistency. For Cervus, the outputs were examined by counting the total number of paternal assignments (including only those with 95% confidence), identifying the total number of fathers for these assignments, and calculating the average Delta (Δ) score (see explanation about this measurement in the main text, section 2.3.2.5) among the high-confidence assignments (Table S1).

In COLONY, similar criteria were tested, but the confidence level was measured using the Maximum-Likelihood score instead of the Δ score used in Cervus (Table S2). All other parameters were defined as described in the main document, section 2.3.2.5. The choice of the final value of the parameter was determined based on the averages of the Δ (Cervus) and Maximum-Likelihood (COLONY) scores produced by both programs. The results of these analyses, presented in Tables S1 and S2, indicate that the highest confidence is achieved when using 70% and 80% as the probability of candidate fathers being present in the sample.

Table S1. The analysis results tested the 'proportion of candidate parents sampled' parameter in the Cervus program.

Proportion of candidate parents sampled (%)	Number of confident paternal assignments	Number of fathers	Average Delta (Δ) score
20	45	22	30.1
30	45	22	31.56
40	45	22	31.56
50	45	25	31.56
60	45	25	31.56
70	45	22	33.5
80	45	22	33.5
90	48	26	33.07

Table S2. The analysis results tested the 'probability of true parents being sampled' parameter in the COLONY program.

Probability of true parents being sampled (%)	Number of paternal assignments	Number of fathers	Average confidence (ML)
20	21	13	0.999997619
30	21	13	0.999997619
40	25	18	0.999997619
50	25	18	0.999997619
60	40	20	0.999997619
70	40	20	1
80	40	20	1
90	40	20	0.999997619

References

Jones, O. R., and J. Wang. 2010. "COLONY: A Program for Parentage and Sibship Inference from Multilocus Genotype Data." *Molecular Ecology Resources* 10 (3): 551-555. https://doi.org/10.1111/j.1755-0998.2009.02787.x.

Marshall, T. C., J. Slate, L. E. Kruuk, and J. M. Pemberton. 1998. "Statistical Confidence for Likelihood-based Paternity Inference in Natural Populations." *Molecular Ecology* 7 (5): 639-655. https://doi.org/10.1046/j.1365-294x.1998.00374.x.
