# Supplementary material for: Resource management as a conservation tool to impact genetic diversity through mating patterns in wild populations
Source: Ecol Appl. 2026 Apr 2;36(3):e70226. doi: 10.1002/eap.70226 (PMC13044502; doi:10.1002/eap.70226)
Supplement: Supplementary file 7 — Appendix S7: [file EAP-36-e70226-s008.pdf]

Appendix S7

Title: Resource management as a conservation tool to impact genetic diversity through mating patterns in wild populations

Authors: Noa Yaffa Kan-Lingwood, Liran Sagi, Alan R. Templeton, Naama Shahar, Ariel Altman, Nurit Gordon, Daniel I. Rubenstein, Amos Bouskila, Shirli Bar-David

Journal: Ecological Applications

Supplementary results for genetic estimates calculation before and during the water source management intervention: observed heterozygosity (H_o) and variance effective population size (N_{ev})

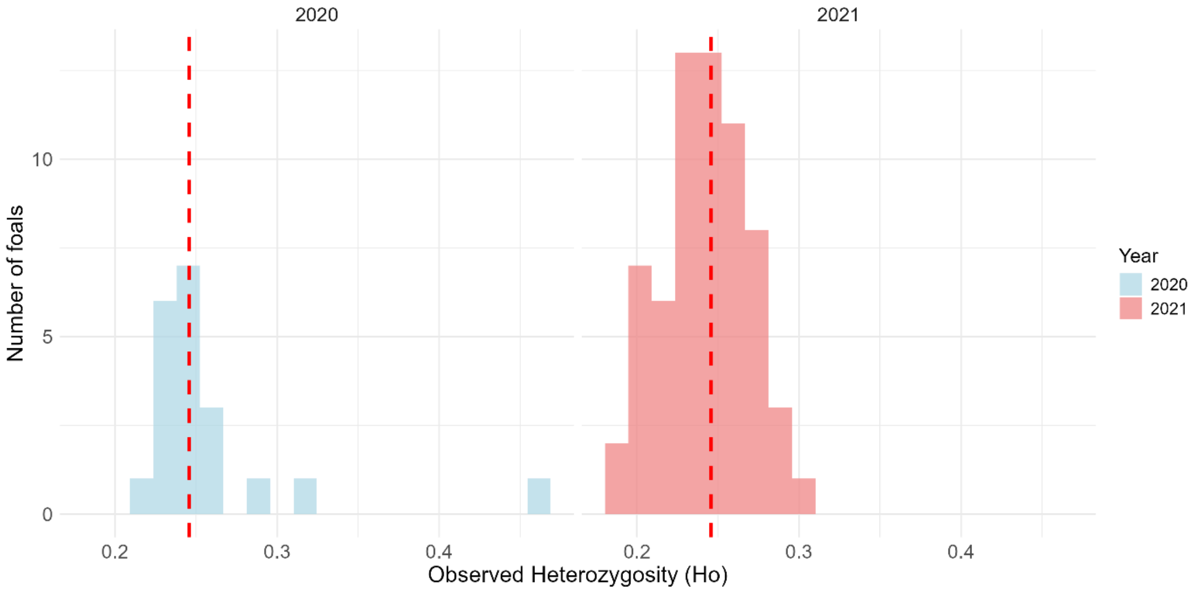

Figure S1. Median H_o test results for foals born in 2020 (left) and 2021 (right). The distribution shows the number of foal individuals with values above and below the pooled median. No significant differences in the median H_o were detected between years (Fisher's exact test: p = 1; KS test: p = 0.308).

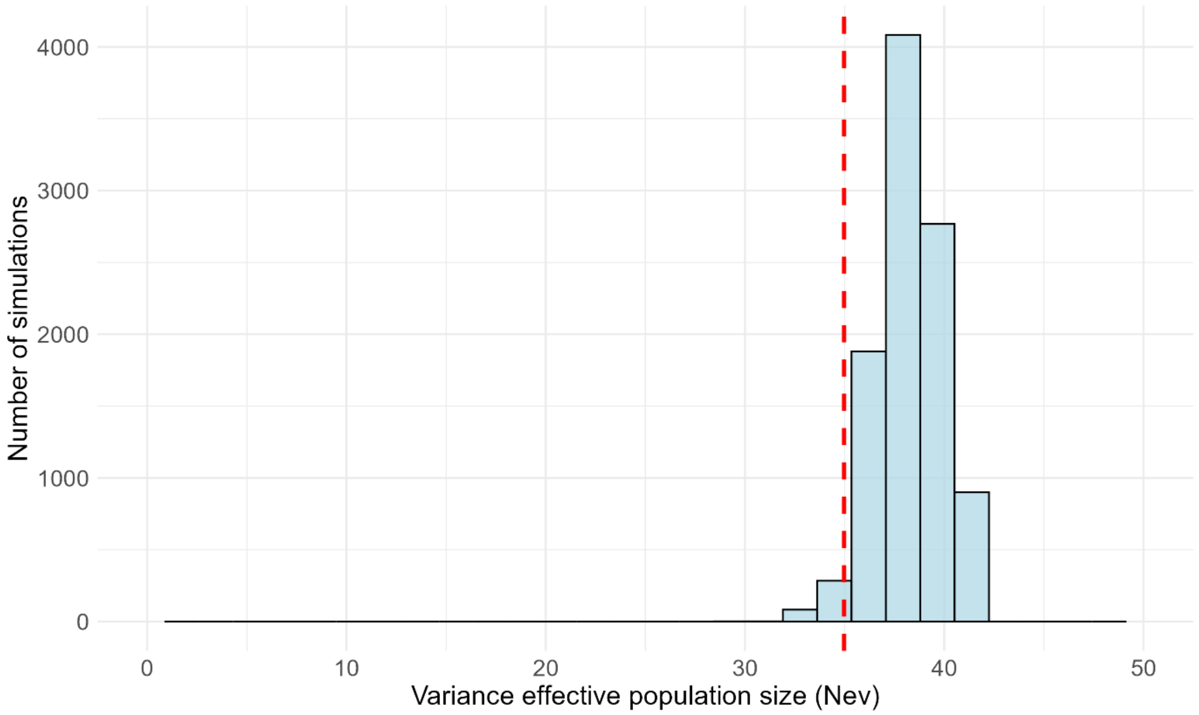

Figure S2. Distribution of resampled variance effective population size (N_{ev}) values for foals born in 2021 (larger sample year; n = 64), subsampled to match the smaller sample size of foals born in 2020 (n = 20; 10,000 simulation runs). In 97.93% of runs, N_{ev} from 2021 foals were higher than the observed N_{ev} from 2020 foals, indicating a significant increase (p = 0.979; two-tailed significance is defined as p < 0.025 or p > 0.975; Walker et al., 2020).

References

Walker, J., M. Mahoney, A. R. Templeton, P. McKenzie, T. E. Vogt, E. D. Cashatt, ... and B. Landwer. 2020. "Contrastsing Ozark and Great Lakes Populations in the Endangered Hines Emerald Dragonfly (*Somatochlora hineana*) Using Ecological, Genetic, and Phylogeographic Analyses." *Conservation Science and Practice* 2 (3): e162. https://doi.org/10.1111/csp2.162
