# Supplementary material for: Resource management as a conservation tool to impact genetic diversity through mating patterns in wild populations
Source: Ecol Appl. 2026 Apr 2;36(3):e70226. doi: 10.1002/eap.70226 (PMC13044502; doi:10.1002/eap.70226)
Supplement: Supplementary file 8 — Appendix S8: [file EAP-36-e70226-s009.pdf]

Appendix S8

Title: Resource management as a conservation tool to impact genetic diversity through mating patterns in wild populations

Authors: Noa Yaffa Kan-Lingwood, Liran Sagi, Alan R. Templeton, Naama Shahar, Ariel Altman, Nurit Gordon, Daniel I. Rubenstein, Amos Bouskila, Shirli Bar-David

Journal: Ecological Applications

Supplemental spatial results

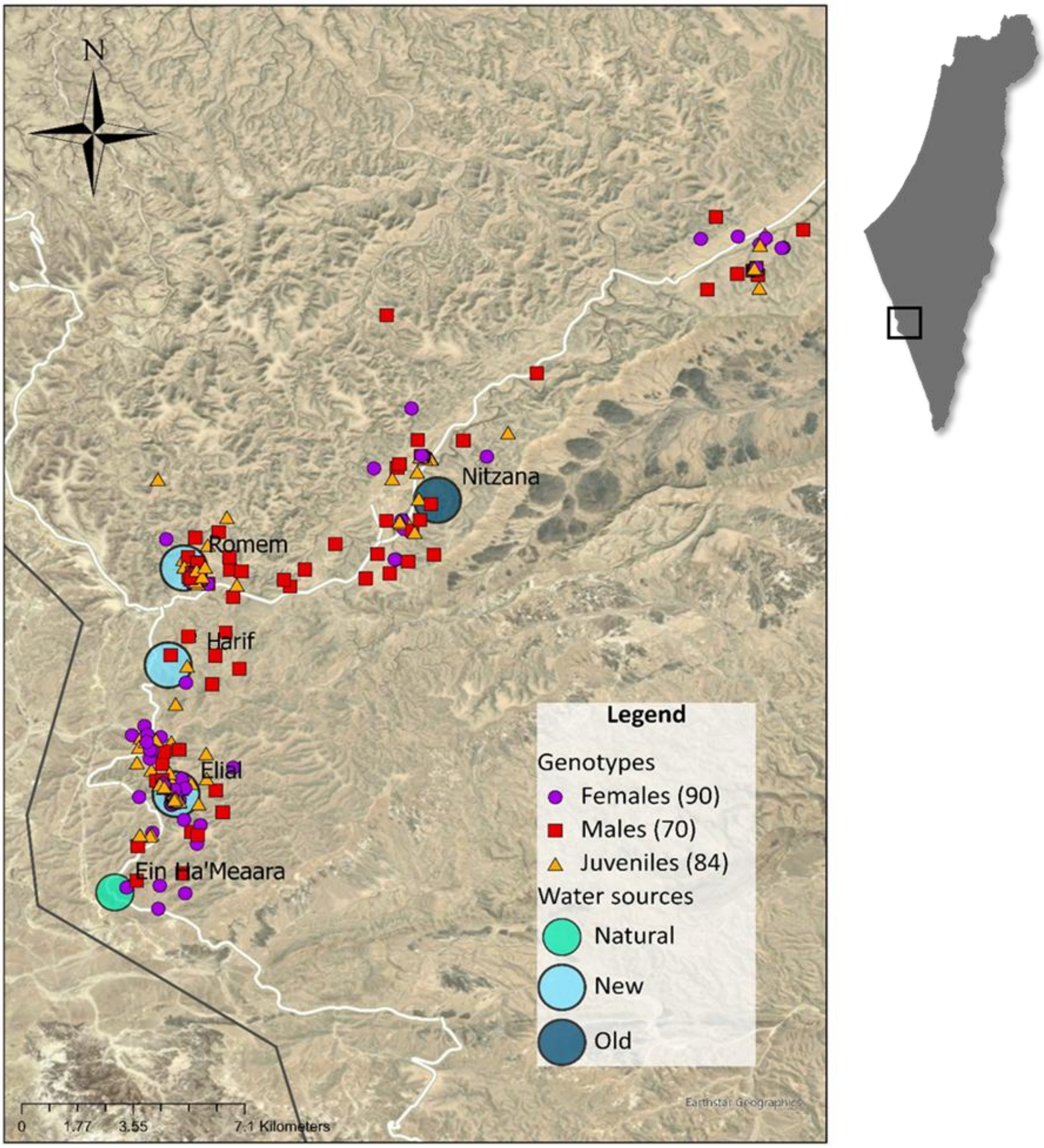

Figure S1. The geographical distribution of 244 unique genotypes of the successful samples collected in the Negev Highlands. One sample location (with the lowest level of missing data) is shown for the cases of recaptured genotypes (Kan-Lingwood et al. 2024).

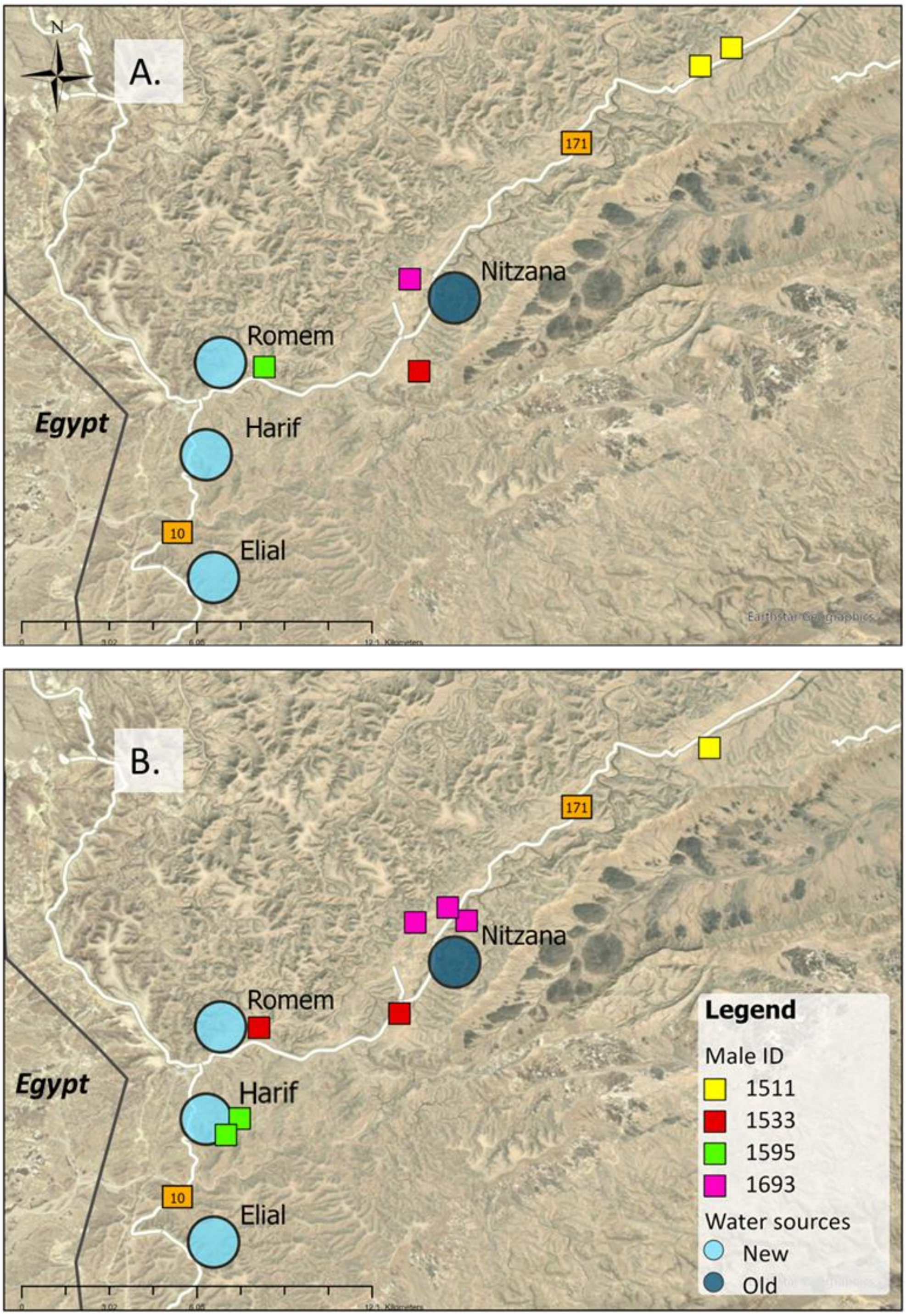

Figure S2. **Spatial recaptures of four adult males that reproduced in both 2019 and 2020 (sires of foals born in 2020 and 2021).** A: Sampling locations of these males in 2020; B: Sampling locations in 2021. Each square color represents a different male ID. Three individuals (IDs 1511, 1533, 1693) were sampled in both years within the same home range. Male 1595, however, was sampled near the new Romem water source in 2020 and later near

the Harif source in 2021, suggesting movement across years and potential establishment of a new territory near a newly accessible water point. These findings support the interpretation of spatial expansion of reproducing males into areas near the new water sources established in 2020.

References

Kan-Lingwood, N. Y., L. Sagi, S. Mazie, N. Shahar, L. Zecherle Bitton, A. Templeton, and S. Bar-David. 2024. "Genotyping Error Detection and Customised Filtration for SNP Datasets." *Molecular Ecology Resources*, e14033. https://doi.org/10.1111/1755-0998.14033.
