# Supplementary material for: Resource management as a conservation tool to impact genetic diversity through mating patterns in wild populations
Source: Ecol Appl. 2026 Apr 2;36(3):e70226. doi: 10.1002/eap.70226 (PMC13044502; doi:10.1002/eap.70226)
Supplement: Supplementary file 9 — Appendix S9: [file EAP-36-e70226-s004.pdf]

Appendix S9

Title: Resource management as a conservation tool to impact genetic diversity through mating patterns in wild populations

Authors: Noa Yaffa Kan-Lingwood, Liran Sagi, Alan R. Templeton, Naama Shahar, Ariel Altman, Nurit Gordon, Daniel I. Rubenstein, Amos Bouskila, Shirli Bar-David

Journal: Ecological Applications

Supplemental parentage simulation results

Running the parental assignments for the simulated genotypes (see sections 2.3.2.4 and 3.1.3 in the main text) with the final dataset of 535 SNPs in 10,000 different replicate analyses, using independent seeds for the random number, showed a similar trend through iterates (individual cycles during the algorithm's execution) and convergence at the final (maximum) likelihood, paternity, and maternity assignments. The average probability across all assignments was 0.999, with a complete assignment of all fictive offspring. Overall, the simulation results show that the final number of SNPs used for the analysis (535) is sufficient and even goes beyond the requirements of a reliable parentage analysis.

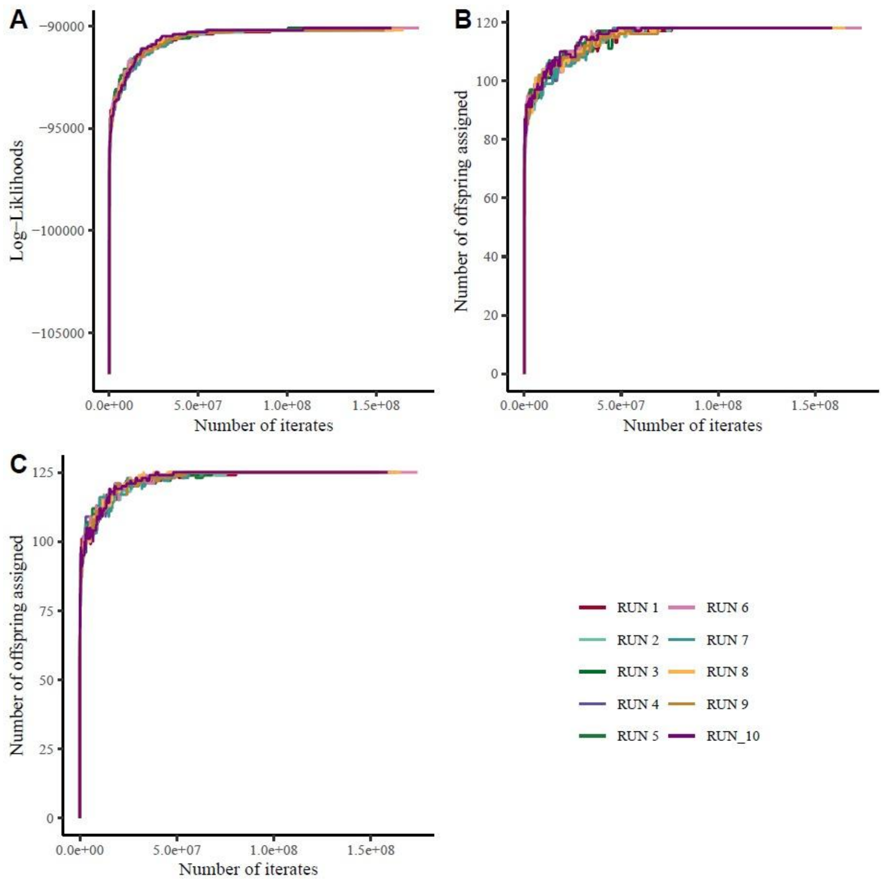

Figure S1. Changes in log likelihoods with increasing iterates of parentage test runs (a), number of inferred paternity assignments (b), and number of inferred maternity assignments (c) with iterates in 10 independent replicate runs. 'Number of Iterates' signifies the progression of the algorithm, tracking how the number of correctly assigned offspring evolves as the program iteratively refines its solution.
