# Supplementary material for: Resource management as a conservation tool to impact genetic diversity through mating patterns in wild populations
Source: Ecol Appl. 2026 Apr 2;36(3):e70226. doi: 10.1002/eap.70226 (PMC13044502; doi:10.1002/eap.70226)
Supplement: Supplementary file 10 — Appendix S10: [file EAP-36-e70226-s001.pdf]

Appendix S10

Title: Resource management as a conservation tool to impact genetic diversity through mating patterns in wild populations

Authors: Noa Yaffa Kan-Lingwood, Liran Sagi, Alan R. Templeton, Naama Shahar, Ariel Altman, Nurit Gordon, Daniel I. Rubenstein, Amos Bouskila, Shirli Bar-David

Journal: Ecological Applications

Table S1. Comparison between paternity results produced by COLONY (pairwise) and Cervus (LOD).

COLONY			Cervus		
Offspring ID	Inferred father	Probability	Offspring ID	Candidate father ID	Pair confidence
2293	1511	95%	2293	1511	Strict (95%)
1536	1515	95%	1536	1515	Strict (95%)
1542	1515	95%	1542	1515	Strict (95%)
1615	1590	95%	1615	1590	Strict (95%)
1890	1590	95%	1890	1590	Strict (95%)
1920	1590	95%	1920	1590	Strict (95%)
1958	1590	95%	1958	1590	Strict (95%)
2327	1590	95%	2327	1590	Strict (95%)
2313	1682	95%	2313	1682	Strict (95%)
1840	1693	95%	1840	1693	Strict (95%)
1518	1771	95%	1518	1771	Strict (95%)
1561	1771	95%	1561	1771	Strict (95%)
2058	1834	95%	2058	1834	Strict (95%)
2212	1834	95%	2212	1834	Strict (95%)
2361	1834	95%	2361	1834	Strict (95%)
1869	1883	95%	1869	1883	Strict (95%)
1906	1883	95%	1906	1883	Strict (95%)
2068	2021	95%	2068	2021	Strict (95%)
2211	2047	95%	2211	2047	Strict (95%)
1560	2048	95%	1560	2048	Strict (95%)
1786	2048	95%	1786	2048	Strict (95%)
1886	2048	95%	1886	2048	Strict (95%)
2228	2048	95%	2228	2048	Strict (95%)
1780	2057	95%	1780	2057	Strict (95%)
2061	2057	95%	2061	2057	Strict (95%)
2139	2057	95%	2139	2057	Strict (95%)
1870	2074	95%	1870	2074	Strict (95%)

2042	2119	95%	2042	2119	Strict (95%)
1541	2264	95%	1541	2264	Strict (95%)
2120	2264	95%	2120	2264	Strict (95%)
1619	2269	95%	1619	2269	Strict (95%)
2233	2269	95%	2233	2269	Strict (95%)
2309	2269	95%	2309	2269	Strict (95%)
2299	2298	95%	2299	2298	Strict (95%)
1934	2305	95%	1934	2305	Strict (95%)
1969	2305	95%	1969	2305	Strict (95%)
2347	2305	95%	2347	2305	Strict (95%)
1864	2340	95%	1864	2340	Strict (95%)
2133	2340	95%	2133	2340	Strict (95%)
1596	6002	95%	1596	6002	Strict (95%)

Table S2. Paternal relationships are inferred by pedigree reconstruction as calculated in the COLONY program (Jones & Wang, 2010) and the sampling year of each offspring and their fathers. Numbers without asterisks represent the IDs of sampled individuals (an offspring or a father). Asterisks (*) represent inferred genotypes of fathers of sampled offspring that were not sampled themselves.

Offspring ID	Year sampled (offspring)	Father ID	Year sampled (parent)
1518	2020	1771	2020
1536	2020	1515	2020
1541	2020	2264	2021
1542	2020	1515	2020
1560	2020	2048	2021
1561	2020	1771	2020
1596	2020	6002 (blood)	2013
1604	2020	*1	-
1615	2020	1590	2020

1619	2020	2269	2021
1667	2020	*2	-
1670	2020	*3	-
1704	2020	*4	-
1711	2020	*5	-
1713	2020	*6	-
1715	2020	*7	-
1780	2020	2057	2021
1781	2020	*8	-
1782	2020	*3	-
1786	2020	2048	2021
1828	2021	*5	-
1831	2021	*9	-
1838	2021	*5	-
1840	2021	1693	2020
1845	2021	*10	-
1853	2021	*11	-
1864	2021	2340	2021
1869	2021	1595	2020
1870	2021	2074	2021
1879	2021	*12	-
1886	2021	2048	2021
1887	2021	*8	-
1890	2021	1590	2020
1901	2021	*6	-
1904	2021	*13	-
1905	2021	*14	-
1906	2021	1595	2020
1920	2021	1590	2020
1924	2021	*15	-
1934	2021	2305	2021
1935	2021	*3	-

1958	2021	1590	2020
1960	2021	*16	-
1965	2021	*1	-
1967	2021	*2	-
1969	2021	2305	2021
1980	2021	*17	-
1995	2021	*13	-
2040	2021	*17	-
2042	2021	2119	2021
2058	2021	1834	2021
2061	2021	2057	2021
2068	2021	2021	2021
2084	2021	*18	-
2085	2021	*5	-
2086	2021	*18	-
2100	2021	*19	-
2120	2021	1533	2020
2133	2021	2340	2021
2139	2021	2057	2021
2150	2021	*20	-
2158	2021	*13	-
2211	2021	2047	2021
2212	2021	1834	2021
2228	2021	2048	2021
2233	2021	2269	2021
2261	2021	*21	-
2285	2021	*22	-
2288	2021	*23	-
2292	2021	*24	-
2293	2021	1511	2020
2295	2021	*24	-
2297	2021	*10	-

2299	2021	2298	2021
2300	2021	*2	-
2302	2021	*25	-
2309	2021	2269	2021
2313	2021	1682	2020
2321	2021	*26	-
2327	2021	1590	2020
2347	2021	2305	2021
2350	2021	*13	-
2353	2021	*27	-
2361	2021	1834	2021

References

Jones, O. R., and J. Wang. 2010. "COLONY: A Program for Parentage and Sibship Inference from Multilocus Genotype Data." *Molecular Ecology Resources* 10 (3): 551-555. https://doi.org/10.1111/j.1755-0998.2009.02787.x.
